# Arthroscopic assisted treatment of distal radius fractures and concomitant injuries

**DOI:** 10.1007/s00402-020-03373-y

**Published:** 2020-03-19

**Authors:** Tobias Kastenberger, Peter Kaiser, Gernot Schmidle, Peter Schwendinger, Markus Gabl, Rohit Arora

**Affiliations:** 1 Department of Trauma Surgery, Medical University of Innsbruck, Anichstrasse 35, 6020 Innsbruck, Austria; 2 Department for Trauma Surgery and Sports Traumatology, Academic Hospital Feldkirch, Carinagasse 47, 6800 Feldkirch, Austria

**Keywords:** Distal radius fracture, Arthroscopic treatment, Concomitant injuries, Intrinsic ligament, Injury

## Abstract

Wrist arthroscopy is mainly used to assist fracture reduction and fixation and to diagnose and treat concomitant injuries mainly to the scapholunate (SL), lunotriquetral (LT) ligament and the triangular fibrocartilage complex (TFCC). Arthroscopy is beneficial in improving anatomical reduction of fracture steps and gaps in intra-articular distal radius fractures (DRFs). Yet, the literature that the functional outcome correlates with the use of arthroscopy, is limited. Non-surgical treatment and immobilization is recommended for Geissler grade I–III Sl-ligament injuries, while open reduction, ligament suture and/or K-wire pinning is mandatory for complete ligament tears according to Geissler grade IV. This manuscript describes the current literature and gives insight into the authors’ opinions and practice.

## Introduction

Distal radius fractures (DRFs) belong to the most common fracture type in humans. Depending on fracture fragment dislocation, fracture instability criteria, the patient’s needs, and functional demands, treatment can vary between non-surgical treatment with cast immobilization and surgical treatment with open reduction and internal fixation. Nowadays, plating is the most common surgical treatment method for DRFs [1]. Wrist arthroscopy in DRFs underwent a cumulative evaluation in the last few decades [2–5]. Over the years, several authors have investigated the benefit of arthroscopy in the treatment of acute DRFs. Indications for arthroscopy in DRFs are multifragmented intra-articular fractures with comminution and/or die-punch fragments, associated carpal bone fractures or obvious intrinsic ligament injuries, an obvious widening of the distal radioulnar joint (DRUJ) suspecting a triangular fibrocartilage complex (TFCC) lesion, and radial styloid fractures because of a potential incomplete greater arch lesion with a SL ligament tear. Wrist arthroscopy is used for diagnostic and therapeutic purposes [6–11].

The following manuscript discusses the current literature regarding these indications and gives insight into the authors` opinions and practice.

## Epidemiology

DRFs are still the most frequent fractures of the upper extremities of patients over 65 years old [12].

Incidence varies topographically. In Scandinavia, the incidence is 30 cases per 10,000 inhabitants per year [13]. Two peaks of prevalence are known: In younger years, at age ten (high energy trauma) and a peak incidence in young patients with a high functional demand [14], and in the elderly at the age of 60 (low energy trauma: fall from standing position). 70% of the fractures occur in women between the ages of 61 and 69 years [15].

### Clinical history

DRFs had been a domain of conservative treatment formerly. During the last few decades, a paradigm shift occurred, from conservative therapy to surgical intervention and follow-up treatment with external fixators and K-wires to volar angular stable plating [13]. Additionally, the use of volar locking plates increased from 42% of plated fractures in 1999 to 81% in 2007 [1].

Why these changes took place cannot be explained, and there is still no evidence for the increase in surgical intervention, especially for angular stable plating systems.

Concerning the changes in our society, fractures are advancing in complexity and patients are increasingly demanding from a functional perspective [5].

### Indications

A main indication for arthroscopy in DRFs is an intra-articular step or gap from 1 to 2 mm after closed reduction [5], which is a prognostic factor for post traumatic osteoarthritis [16, 17]. Arthroscopically assisted treatment of DRFs can help detecting and treating scaphoid fractures and/or ligament injuries. Radiographic findings may hint to soft tissue injuries e.g., inter-carpal joint space widening or disruption of the Gilula lines.

Radial styloid fractures (chauffeurs fracture) without the dislocation of the lunate may be part of greater arch injuries described by Mayfield [18].

Widening of the DRUJ may hide an injury of the TFCC, which can be verified arthroscopically.

Complex multifragmented intra-articular fractures such as three-part/four-part fractures associated with intra-articular comminution (explosion type fractures) may need arthroscopic evaluation, reduction, and fixation, and in die-punch fractures, arthroscopy is suggested too [10]. Few authors even recommend wrist arthroscopy for any kind of DRF [19].

The authors perform arthroscopy in the following cases:

- Intra-articular DRFs with a sagittal fracture line mainly at the height of the scapholunate ligament to evaluate any concomitant scapholunate ligament injury.
- Intra-articular DRF with a sagittal or frontal fracture line with larger relevant fragments for fracture reduction to minimize fracture steps and gaps (e.g., Die punch fragments).
- Intra-articular DRFs with an impacted central fragment to evaluate correct fragment reduction.
- Intra-articular DRFs with an unstable, DRUJ for TFCC assessment [20].
- Galeazzi type DRFs with a loss of radial length of > 6 mm.
- DRFs with radiologic predictors of DRUJ instability (radial inclination < 11°, dorsal tilt > 20°, radioulnar interval > 2 mm, base fractures of the ulnar styloid process).
- DRFs with scapholunate interval opening during dynamic intraoperative fluoroscopic evaluation with radial- and ulnar abduction.

### Contraindications

Low-activity patients, extra-articular fractures in the elderly, open fractures, and DRFs associated with other multiple fractures are reported to be contraindications for wrist arthroscopy [19].

### Wrist arthroscopy setup

Although simple diagnostic wrist arthroscopy and soft tissue procedures can be performed under wide awake local anesthesia and no tourniquet (WALANT) surgery [21], patients with intra-articular DRFs are usually treated under general or regional anesthesia, in case of the need for manipulation of fracture fragments, which might be painful [19].

**Fig. 1:**
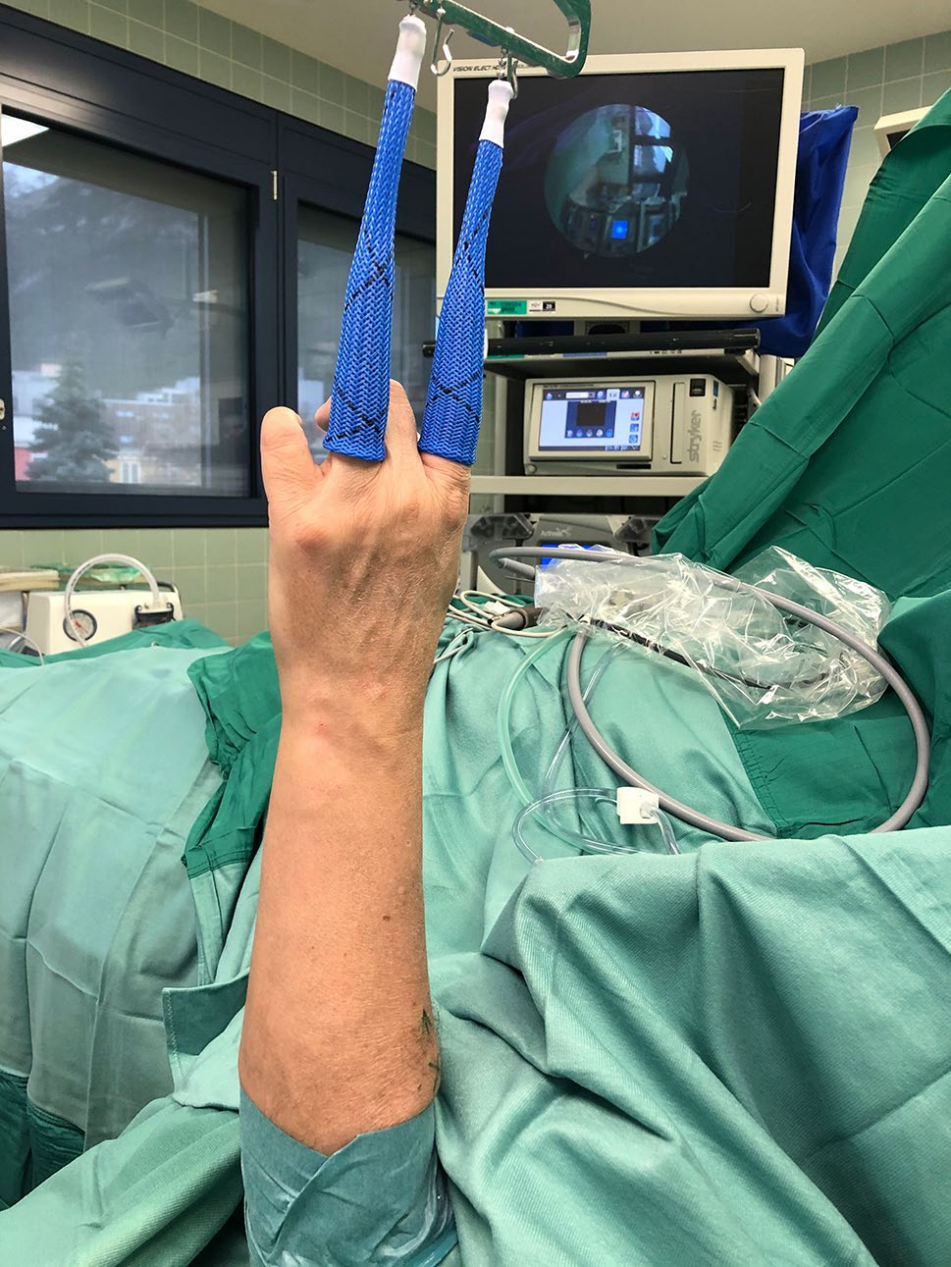
Patient lying in a supine position with the upper extremity suspended by the use of finger traps

The patients are in supine position with the fingers hanging by the use of extension sleeves (Fig. 1).

**Fig. 2:**
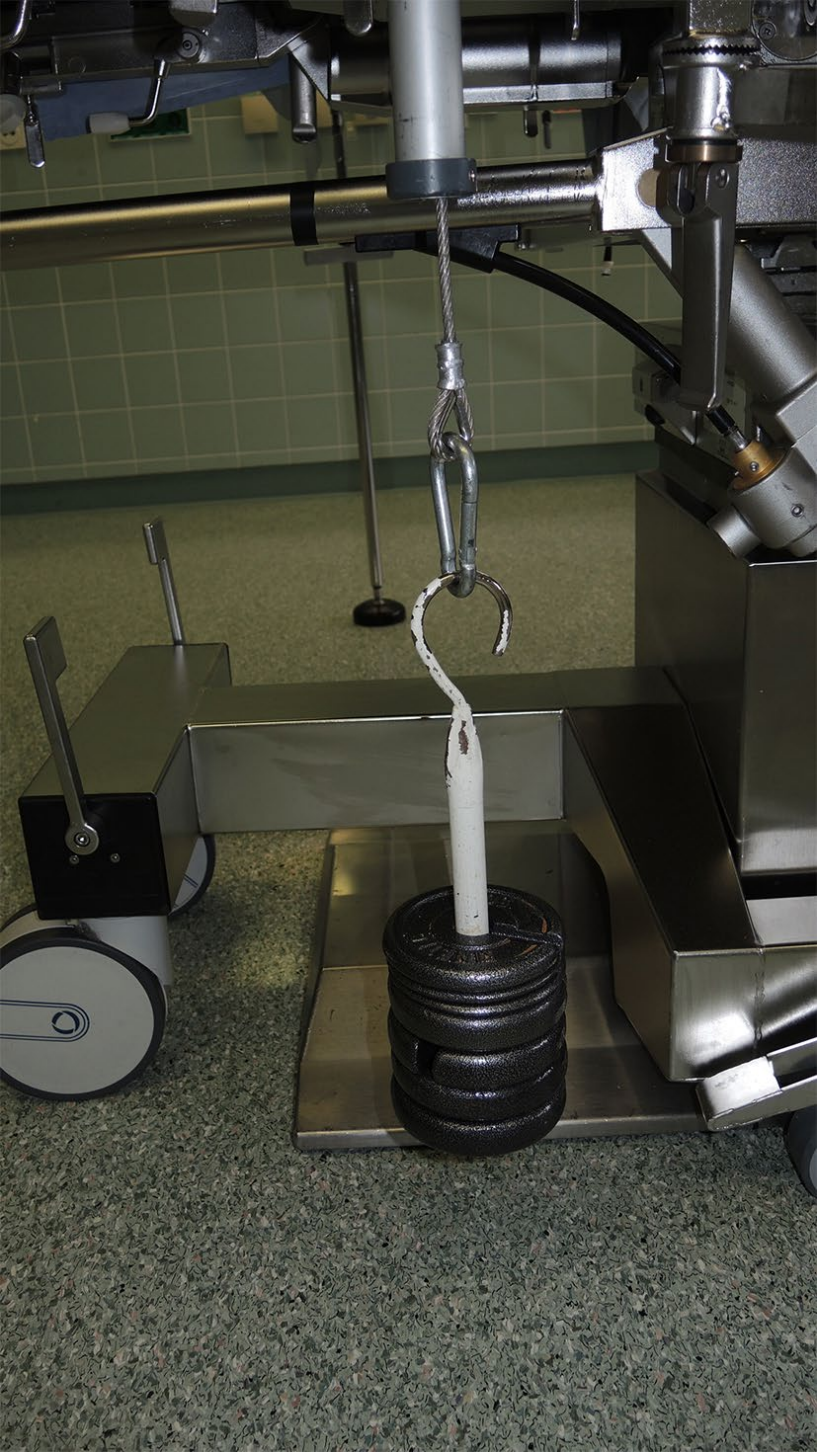
Four kg traction weight applied on the wrist

Traction is applied by dead weights (approximately 3–4 kg) (Fig. 2).

An Esmarch tourniquet on the arm is inflated for exsanguination to 200–250 mm Hg [19].

The authors usually use “dry” arthroscopy according to the recommendations of Del Pinal [22] to prevent extravasation, to minimize soft tissue swelling and secondary compartment syndrome [19, 22]. In “dry” wrist arthroscopy, the air valve is kept open to enable free air circulation through the joint and suction should be switched off unless needed. One disadvantage is the potential loss of vision due to splashes on the tip of the scope, or blood and debris in the joint. If required, the joint can be irrigated using saline to remove debris and blood to increase visibility. The surgeon should switch to “wet” arthroscopy when using thermal probes because heat generation may damage the cartilage [22]. If needed, a pump pressure of 15 mmHg should be sufficient [5].

**Fig. 3:**
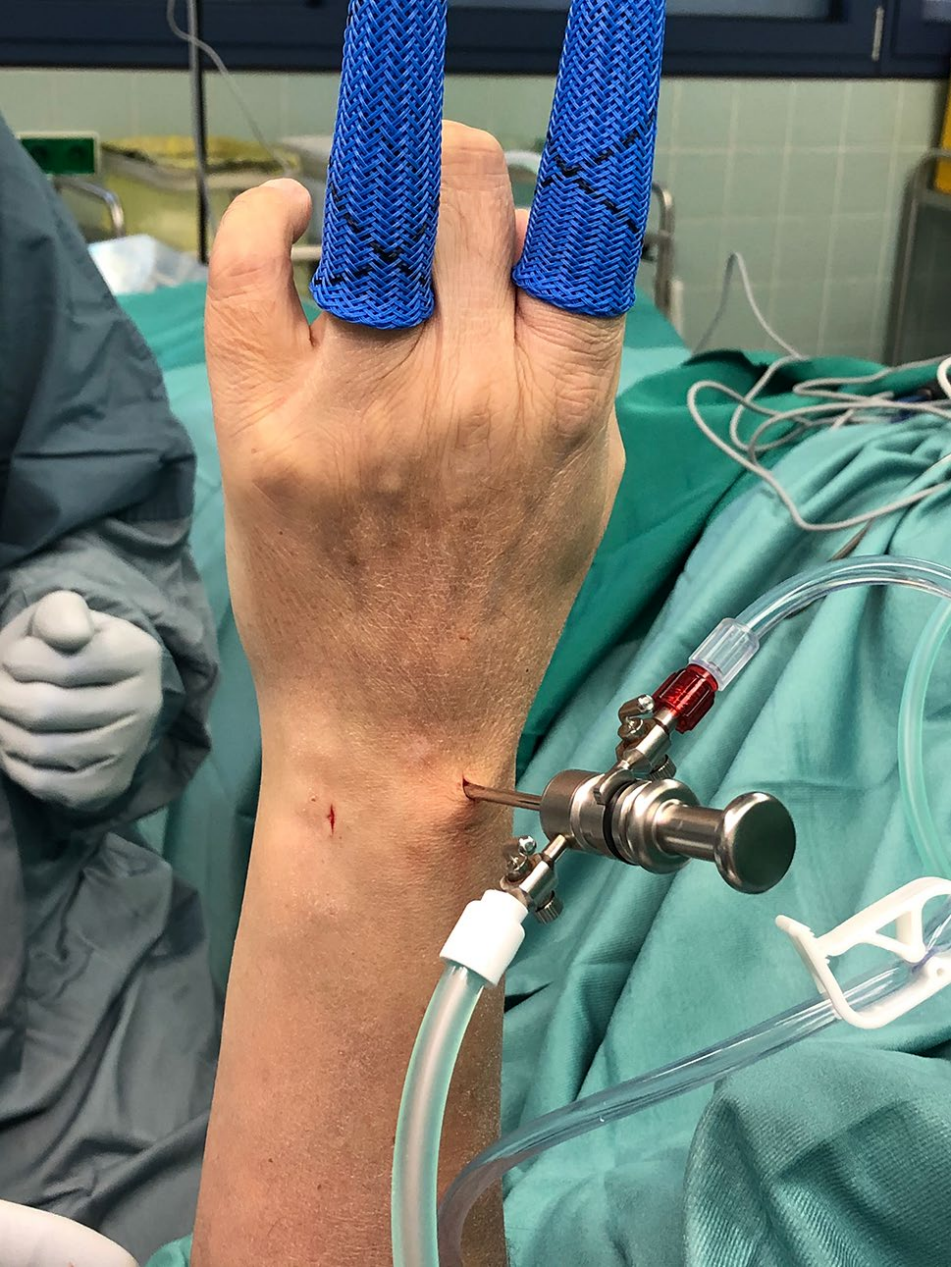
Standard arthroscopy portals 3/4, and 6R with the camera in the 3/4 portal

The authors use a 2.7 mm probe with a 30° field of view angle. The radiocarpal joint is typically assessed using the 3/4, 5/6 and 6R portal, and the midcarpal joint using the midcarpal radial and ulnar portals (Fig. 3).

Any fracture steps or gaps, chondral lesions, and the TFCC itself are assessed via the radiocarpal portals, whereas the SL and LT ligaments are assessed via the midcarpal portals.

Before using arthroscopy as a standardized procedure, experience is required, to prevent prolongation of surgical time, as well as an increase in iatrogenic injury risks (nerve and tendon lacerations). Arthroscopy shows an exponential learning curve with a reduction of complication rates by time with ongoing surgeon experience, and a threshold at over 5-year expertise [5, 23].

### Assisted fracture reduction

Intra-articular DRFs should be reduced anatomically without any persisting steps, as the latter correlates with radiocarpal osteoarthritis and an unsatisfactory result [16]. Recent findings indicate a critical tolerance for joint incongruity in the distal radius may be as little as 1 mm [24–27].

Therefore, it seems reasonable to invest all effort into adequate intra-articular fracture reduction. Arthroscopy is used to directly visualize any intra-articular gaps and step-offs and fracture reduction under visualization.

Reports with good illustrations showing the technique of arthroscopic fracture reduction are published by Del Pinal et al. [28], Ardouin et al. [5], and Lutz et al. [29]. Arthroscopy can be performed either before or after initial fracture reduction [30]. We initially performed an extra-articular fracture reduction of the metaphysis and fixation of the reduced fragments using a plate that is fixed at the radius shaft. Ligamentotaxis and indirect manipulation of intra-articular fracture fragments using the “joysticks reduction technique of the joint surface” is performed under fluoroscopic control. Intra-articular fracture fragments are temporarily stabilized using K-wires [31].

The plate is fixed through its gliding hole in the diaphysis to ensure a more distal or proximal plate position. Additional K-wires can be inserted distally to stabilize the reduced intra-articular fragments [32].

**Fig. 4:**
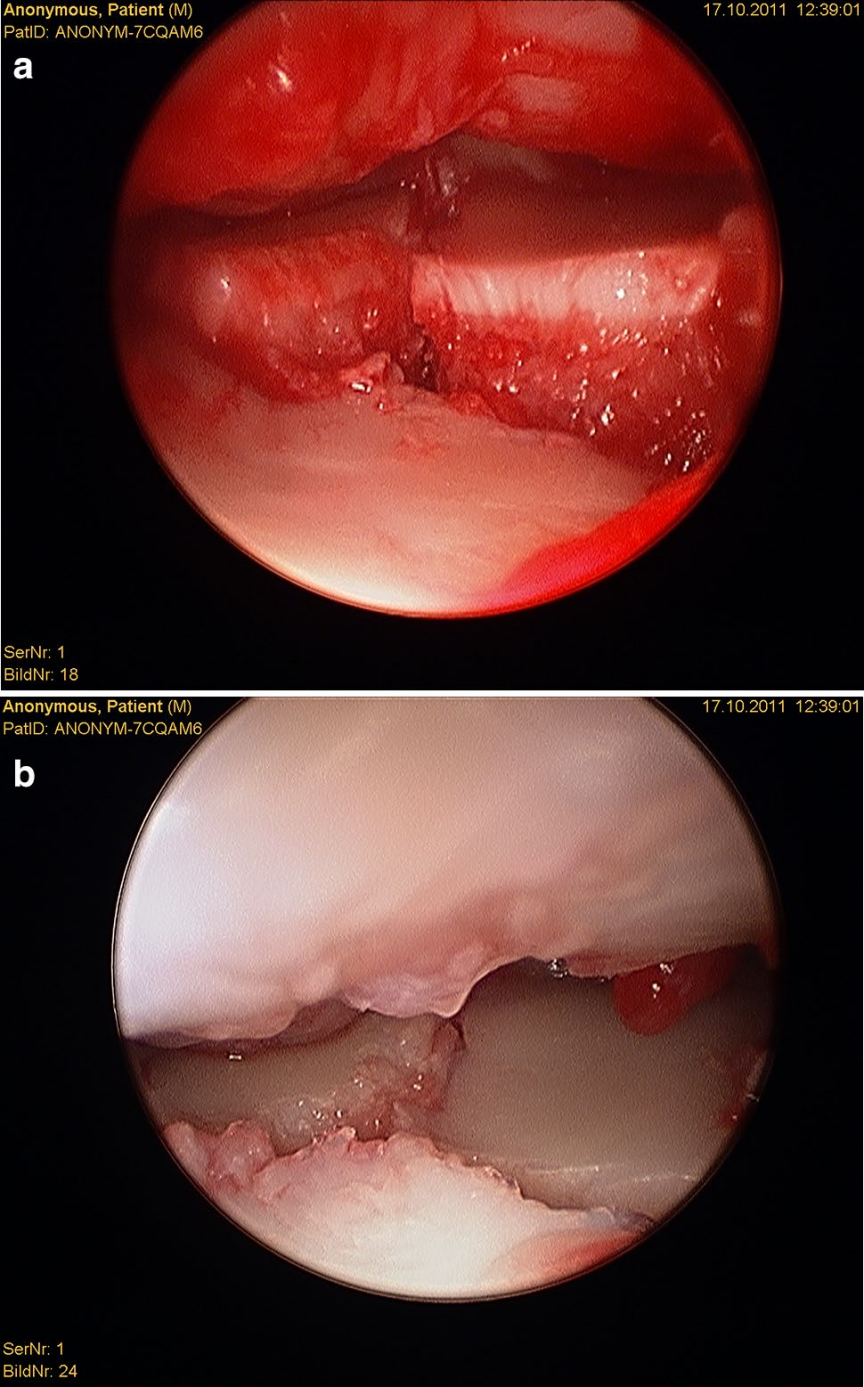
Radiocarpal view **a** before fracture reduction and **b** after fracture reduction

After plate presetting, the forearm is placed in a vertical traction tower and extension weight is applied. One longitudinal incision is placed at the 3/4 portal between the extensor pollicis longus tendon and the extensor digitorum communis tendon. A 2.7 mm arthroscope is inserted to inspect the joint. A second longitudinal incision is placed at the 6R portal and a probe is inserted which can be used to manipulate the intra-articular fragments (Fig. 4a, b). The remaining hematoma should be removed for better visualization.

In cases of increased steps or gaps, the K-wires are driven back and an improvement of intra-articular reduction is performed under direct arthroscopic visualization. As long as the fracture is only temporary reduced and fixed using K-wires, every step and gap can be addressed by removing a particular K-wire to loosen that specific fragment. The reduction of this fragment is conducted under direct visualization using a hook probe. The loosened K-wire is introduced through the fracture fragment for temporary fixation. Additionally, traction through the finger traps helps to ensure radial inclination.

The joint surface and the intrinsic ligamentous injuries are inspected from the 3–4 portal arthroscopically. Debridement of joint cartilage fragments, fracture hematoma, and early granulation tissue can be performed using a shaver [19].

Once the reduction has been completed successfully, the epiphyseal screws are positioned through the plate drill holes, and the K-wires are removed. One last joint inspection after distal screw positioning is performed checking the absence of intra-articular screw protrusion and intra-articular fragment stability.

Finally, a fluoroscopy is used to check the screw length in an anterior- posterior, lateral, and dorsal-horizontal view.

Several studies have been conducted to address the question whether fluoroscopically or arthroscopically assisted reduction leads to a better articular surface restoration regarding the size of steps and gaps. A systematic review including 720 patients with a follow-up of 0–38 months (16 studies; level of evidence II–IV) showed that most studies (13/16) were in favor of arthroscopy, improving articular reduction [33].

However, most studies had a small sample size, short follow-up and/or used a case-series design, which is unable to answer the question of articular reconstruction [33]. Furthermore, the correlation of anatomical reconstruction with the clinical and functional outcome, that has more importance for the treated patients, could not be answered in any of the studies.

Catalano et al. and Goldfarb reported that short and long-term functional outcomes did not correlate with the magnitude of the residual step-off and gap displacement, with the awareness that most of the patients had a good restoration of extra-articular alignment with surgical treatment and few had major articular incongruities [27, 34–36].

Regarding the question whether arthroscopy helps to improve functional scores at last follow-up, Saab et al. reported that only 50% of the studies (6/12 studies; level of evidence II–IV) were in favor of arthroscopy. However yet again, most studies had a small sample size, short follow-up and/or used a case-series design [33].

Arthroscopy allows us to visualize acute chondral lesions of the distal radial joint surface which can be seen as subchondral hematomas, cracks, avulsed cartilage flakes, or complete avulsions of the cartilage [10]. Subchondral hematoma may lead to osteoarthritis as well as destroyed cartilage [37]. Currently, debridement appears to be the only available option, although micro fracturing may be attempted without adequate proof of its benefit [10]. Yet, severely comminuted radius fractures with destroyed cartilage may even need, or benefit from, partial wrist fusion or hemi-arthroplasty instead of arthroscopically assisted open reduction and internal fixation [10, 36, 38–41].

**Fig. 5:**
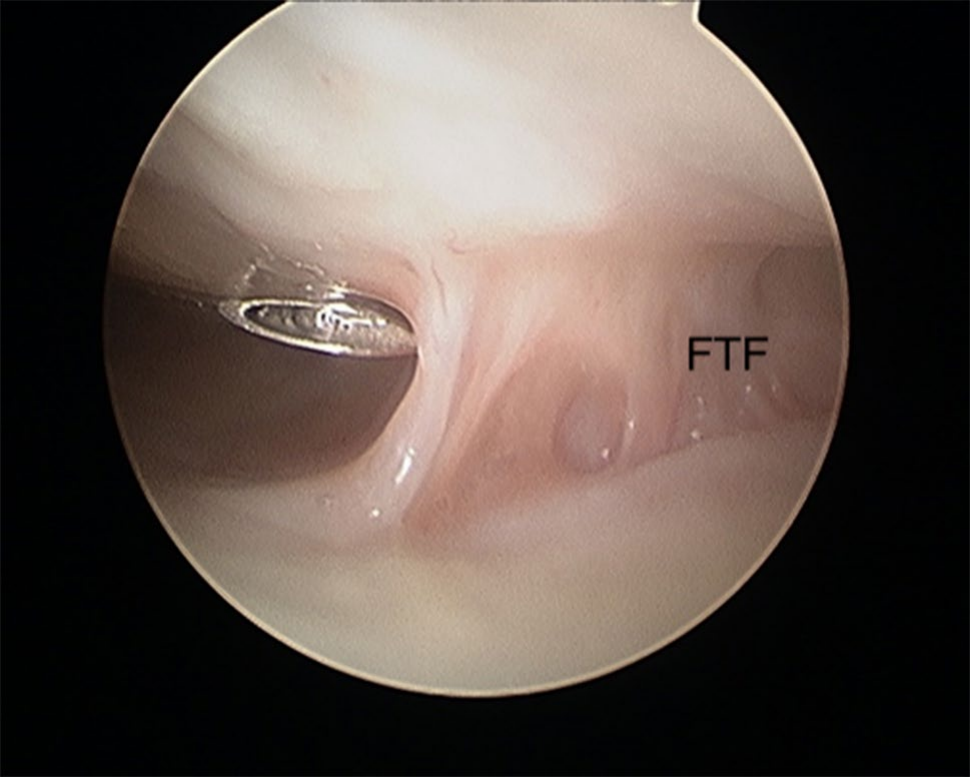
Intra-articular fibrous tissue formation (FTF)

**Fig. 6:**
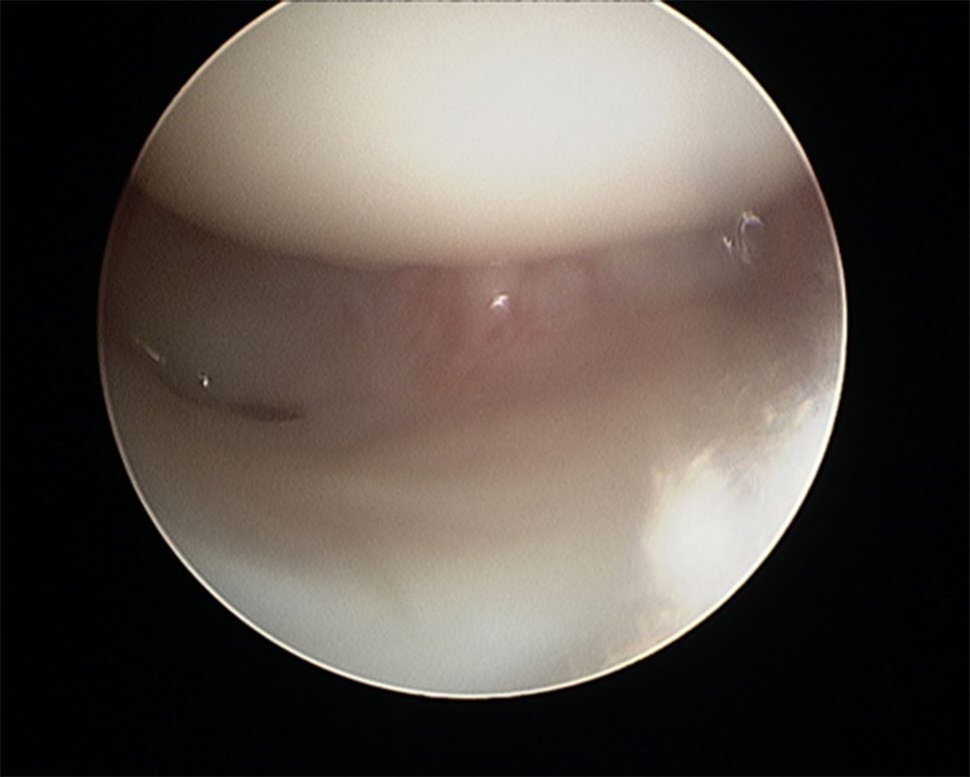
Intra-articular view after debridement of FTF

Gabl et al. [42] investigated the formation of post-operative intra-articular fibrous tissue in DRFs which potentially causes loss of radio-carpal motion (Figs. 5 and 6). The author found that fibrotic tissue develops and extends from former fracture gaps to the SL ligament and the dorsal joint capsule, while undamaged cartilage remains without fibrous tissue formation. All fibrous tissue was too tight to be removed without shaving and potentially limited the range of motion of the proximal carpal row [42]. Therefore, arthroscopic debridement after fracture healing at the time of implant removal of these intra-articular fibrous scar tissues may improve the range of motion and functional scores.

Finally, it seems that arthroscopic removal of intra-articular hematoma and debris during primary surgical fracture fixation did not show any benefits regarding functional outcome in comparison to patients treated via open reduction and internal fixation alone [43].

### Concomitant soft tissue injuries

The authors adapt to the circumstances of the surgery regarding the order of investigation of further concomitant injuries, which are mainly TFCC, SL and LT ligament injuries. They are assessed either before or after definite fracture reduction and fixation.

Concomitant injuries to the SL ligament occurs in about 30–50% [5, 44–46]. A systematic review described a lesion of the scapholunate interosseous ligament in 41% (12 studies; 467 patients; mean follow-up 22 months). 76.2% were classified as Geissler grade I or II and 23.8% as Geissler grade III or IV.

LT injuries are reported in 8.5–15% [44, 47] and TFCC injuries in more than 50% with the majority showing degenerative origin [5, 10, 46].

### SL and LT ligament injuries

**Fig. 7:**
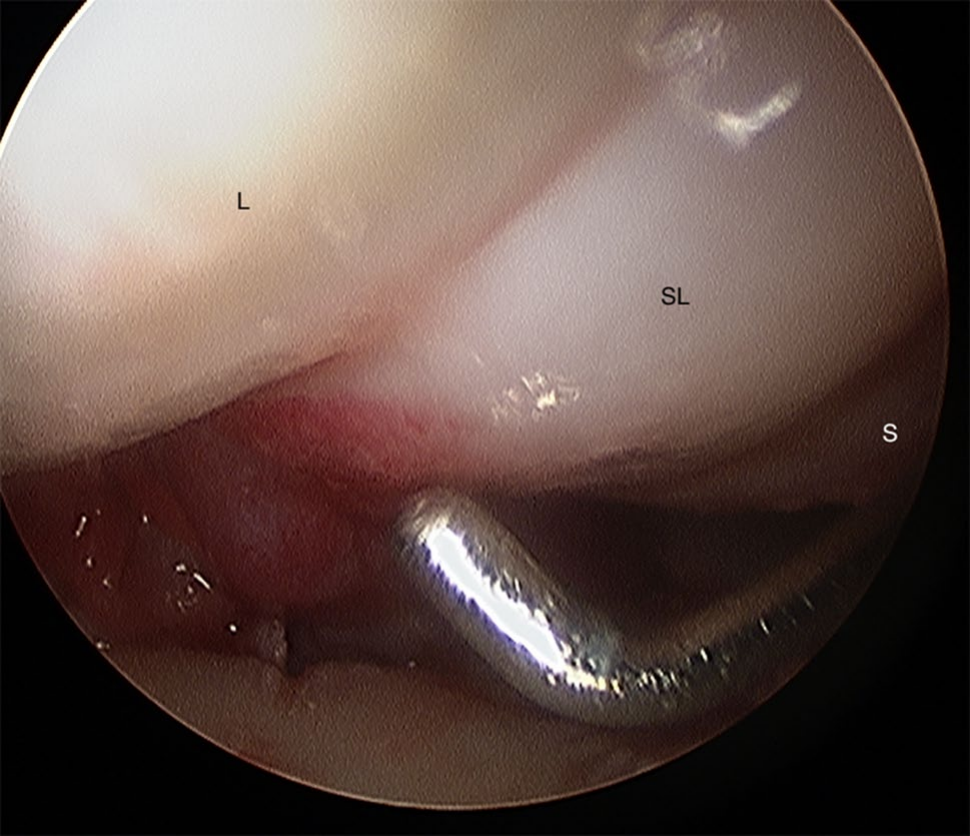
Radiocarpal view: balloon sized scapholunate ligament Geissler grade 1; *L* lunate, *SL* scapholunate ligament, *S* scaphoid

**Fig. 8:**
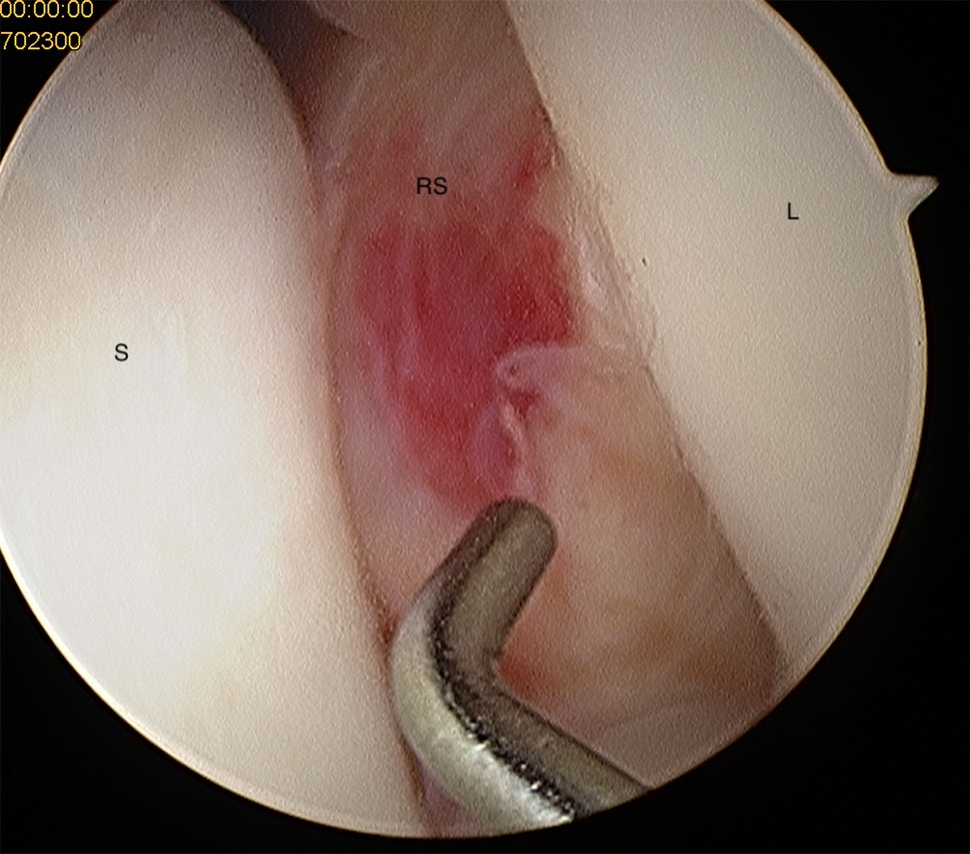
Midcarpal view: radius joint surface can be seen with the hook between scaphoid and lunate indicating a scapholunate ligament tear Geissler grade 4; *S* scaphoid, *RS* radial joint surface, *L* lunate

It is important to evaluate SL and LT ligament injuries associated with DRFs, as these can be part of incomplete greater arch injuries as described by Mayfield et al. [18]. These injuries can be directly visualized and classified as partial or complete along their dorsal, membranous, and palmar parts [10] (Figs. 7 and 8). The degree of injuries can be classified according to Geissler [48] (Table 1) or the latest EWAS (European Wrist Arthroscopy Society) classification (Table 2). The latter EWAS classification will be used in future scientific evaluations, however, because most of the cited studies used the Geissler classification, the current manuscript also reports the treatment suggestions according to the Geissler classification. The widening of inter-carpal joint spaces reflect the degree of mobility/instability of the affected joint as a consequence of ligamentous injury.

**Table 1:** Geissler classification [40]

Grade	Description
Geissler I	Hemorrhage of the interosseous SL ligament seen from the radiocarpal joint. No incongruence of carpal alignment in the midcarpal space
Geissler II	Hemorrhage of interosseous SL ligament seen from the radiocarpal joint. Incongruence/step-off seen from the midcarpal space. A slight gap (less than the width of a probe) between carpals may be present
Geissler III	Incongruence/step-off of carpal alignment seen in both the radiocarpal and midcarpal spaces. The probe may be passed through the gap between carpals
Geissler IV	Incongruence/step-off of carpal alignment seen in both the radiocarpal and midcarpal spaces. Gross instability with manipulation. A 2.7-mm arthroscope may be passed through the gap between carpals from the midcarpal space

**Table 2:** Arthroscopic EWAS (European Wrist Arthroscopy Society) Classification

Arthroscopic stage (EWAS)	Arthroscopic testing of SLIOL from MC joint
I	No passage of the probe in SL space but synovitis
II	Lesion of membranous SLIOL Passage of the tip of the probe in the SL space without widening (stable)
III A	Partial lesion involving the volar SLIOL Volar widening on dynamic testing from MC joint (anterior laxity)
III B	Partial lesion involving the dorsal SLIOL Dorsal SL widening on dynamic testing (posterior laxity)
III C	Complete SLIOL tear, joint is reducible Complete widening of SL space on dynamic testing, reducible with removal of probes
IV	Complete SLIOL with SL gap SL gap with passage of the arthroscope from MC to RC joint No radiographic abnormalities
V	Wide SL gap with passage of the arthroscope through SL joint Frequent X-ray abnormalities such as an increased SL gap, DISI deformity

*SLIOL* scapholunate interosseous ligament, *MC* midcarpal, *RC* radiocarpal, *RSC* radio-scapho-capitate, *LRL* long radiolunate, *DIC* dorsal intercarpal ligament, *SL* scapholunate, *TH* triquetro-hamate, *ST* scaphotrapezial, *DRC* dorsal radiocarpal, *DISI* dorsal intercalated segmental instability

These injuries have the potential to proceed to SL dissociations and secondary carpal instability if left untreated [7], and eventually lead to posttraumatic scapho-lunate advanced collapse (SLAC) osteoarthritis [10]. It is important to detect and properly treat tears early to avoid long-term problems.

Geissler suggested immobilization of grade I injuries, arthroscopic reduction, and K-wire pinning for grade II injuries, arthroscopic or open reduction and K-wire pinning for grade III injuries, and open reduction and repair for grade IV injuries [48].

This approach seems too aggressive because patients with low grade incomplete tears (grade I and II) are asymptomatic at 1 year after DRF surgery [7] if only immobilized. There were no long-term findings for developing a SLAC wrist for grade I and II [49]. The immobilization protocol after palmar plating needs to be adapted, depending on the grade of SL injury [50].

The treatment of grade III injuries remains controversial. Several authors besides Geissler advocate K-wire pinning [33, 51–55]. However, there were no differences found in the subjective, objective, or radiographic outcome after grade III and grade I/II untreated tears associated with displaced DRFs [49]. The reason for such findings may be the secondary stabilizers of the wrist.

Acute grade IV injuries show a dynamic instability and need surgical treatment using arthroscopic or open reduction and K-wire pinning and/or anchor fixation [10]. Typically, the scapho-lunate and the scapho-capitate joints are transfixed using K-wires. Additional cast fixation is needed for 8–10 weeks until the K-wires are removed.

**Fig. 9:**
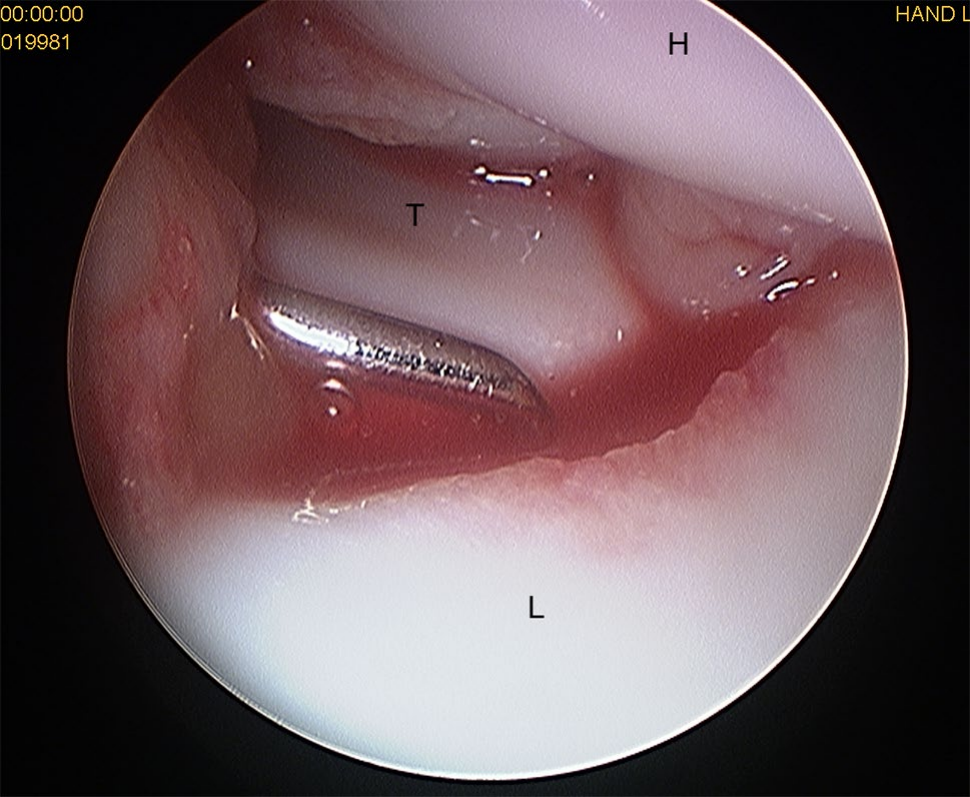
Midcarpal view: widening of the lunotriquetral joint with the hook between the triquetrum and lunate indicating a lunotriquetral ligament tear; *T* triquetrum, *L* lunate, *H* hamate

A similar approach is recommended for LT injuries (Fig. 9) and is also used by the authors. Stable grade I, II, and III injuries are immobilized for up to 4 weeks depending on the stability of fracture fixation and bone quality, and grade IV injuries need arthroscopic debridement and percutaneous K-wire pinning with an immobilization for 8 weeks [10].

However, the evidence of this approach is limited. There are no long-term studies investigating the impact of cast fixation in partial injuries. Additionally, it is questionable if the improved diagnosis using arthroscopy, and the resulting treatment of lower graded injuries leads to a better functional outcome. Swart and Tang [55] did not find any major differences in the subjective and objective outcome measurement in patients with and without ligament injuries.

### TFCC injuries

The importance of TFCC tears and DRUJ instability is explained in a separate chapter within this journal. Therefore, the authors will not discuss any details in this manuscript [20].

### Complications

At least the complication rate following wrist arthroscopy is low. The most reported complications of wrist arthroscopy are: procedure failure 1.16% and nerve injuries 1.17%; tendon lacerations are less frequent [23].

Other complications include a loss of reduction, loosening of a K-wire, pin infections, complex regional pain syndrome, cartilage lesions, joint stiffness, and remaining loose bodies [23, 56, 57].

## Conclusion

Wrist arthroscopy appears to be beneficial in diagnosing intra-articular steps and gaps, as well as concomitant injuries to the scapholunate and lunotriquetral ligament, and the triangular fibrocartilage complex in DRFs. It allows any step-off or gap malalignment to be addressed via direct visualization. The author`s indications for wrist arthroscopy in DRF’s are intra-articular fractures with a sagittal fracture line at the level of the scapholunate ligament to exclude a scapholunate ligament injury, intra-articular DRF with a sagittal or frontal fracture line with larger relevant fragments for fracture reduction to minimize fracture steps and gaps (e.g., Die punch fragments), intra-articular DRF with an impacted central fragment to evaluate correct fragment reduction, intra-articular DRF with an unstable DRUJ for TFCC assessment and DRF with a scapholunate interval opening during dynamic intraoperative fluoroscopic evaluation with radial and ulnar abduction. For the authors, non-surgical treatment and immobilization for up to 4 weeks is recommended for Geissler grade I–III SL and LT injuries because the effort of arthroscopically conducted ligament repair has to be regarded critically in these cases as patients’ benefits have not been proven. Open reduction, ligament suture, and/or K-wire pinning is mandatory for complete ligament tears Geissler grade IV.

However, to date, there is limited evidence that arthroscopy shows benefits in clinical outcome parameters. Prospective long-term studies are needed to confirm the need and beneficial role of arthroscopy and the proposed treatment regime in DRFs as well as SL and LT injuries.

### Clinical case

**Fig. 10:**
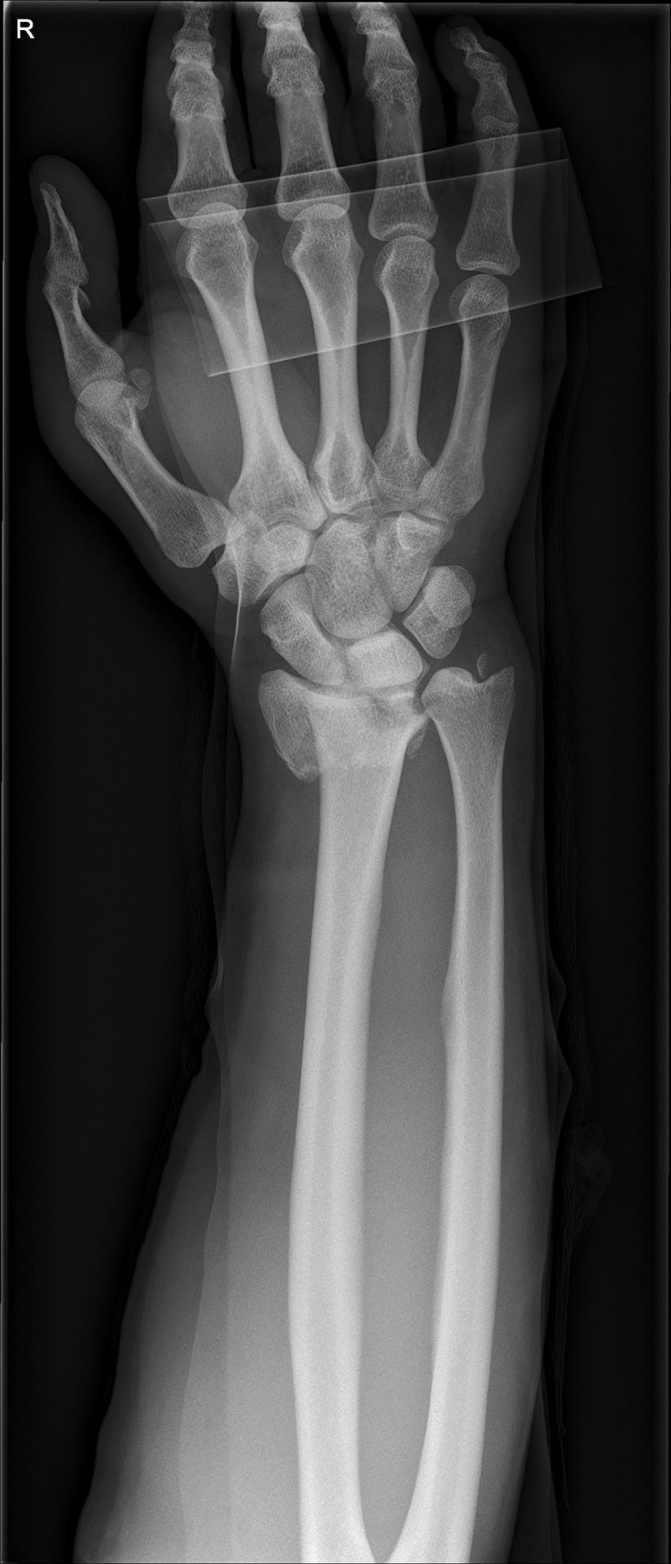
Dorsovolar view: initial assessment of the injured wrist with a loss of the radial inclination and radial length

**Fig. 11:**
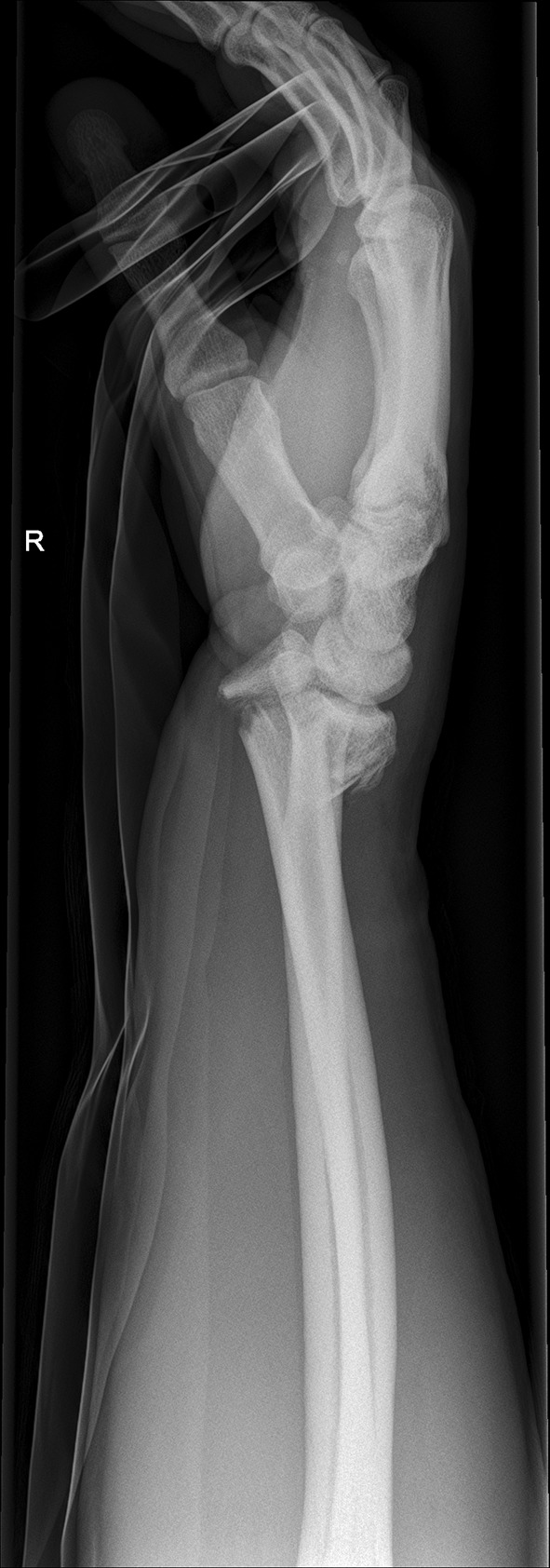
Lateral view: initial assessment showing the loss of radio-ulnar and dorso-palmar inclination

We present a case of a male patient aged 31 years who sustained an intra-articular four-part DRF in 2011. The main instability area is located at the intermediate column. The initial antero-posterior (Fig. 10) and lateral (Fig. 11) X-rays show a loss of the radial length, radio-ulnar and dorso-palmar inclination.

**Fig. 12:**
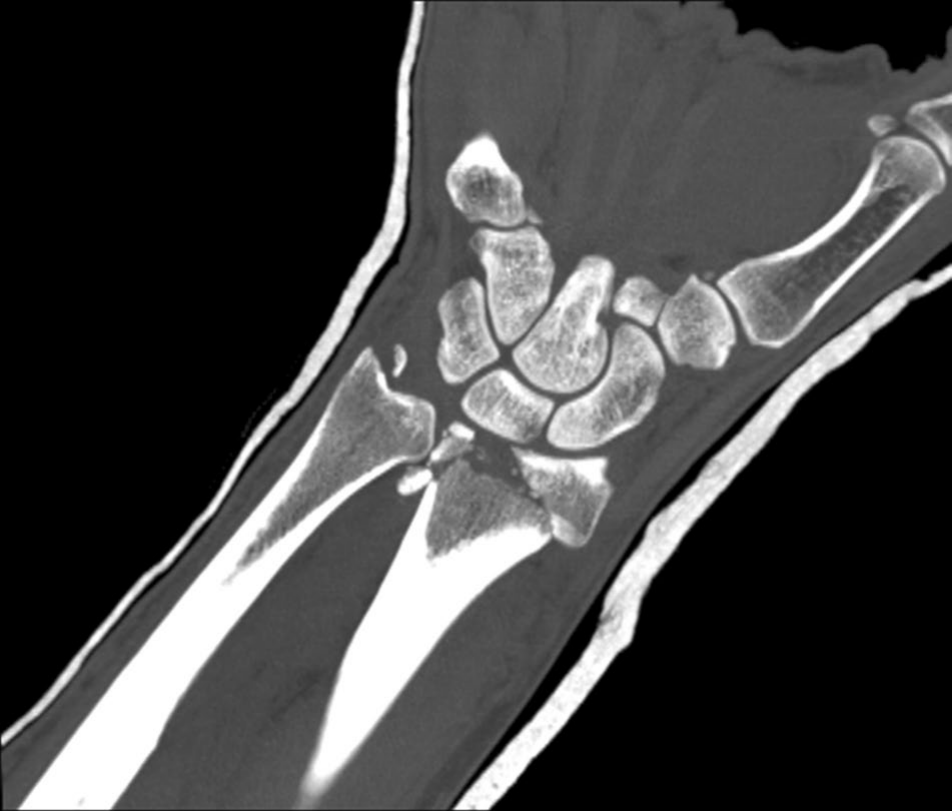
CT scan of a dorsovolar view after reduction and cast immobilization showing the defect zone of the intermediated column and fracture involvement of the DRUJ

**Fig. 13:**
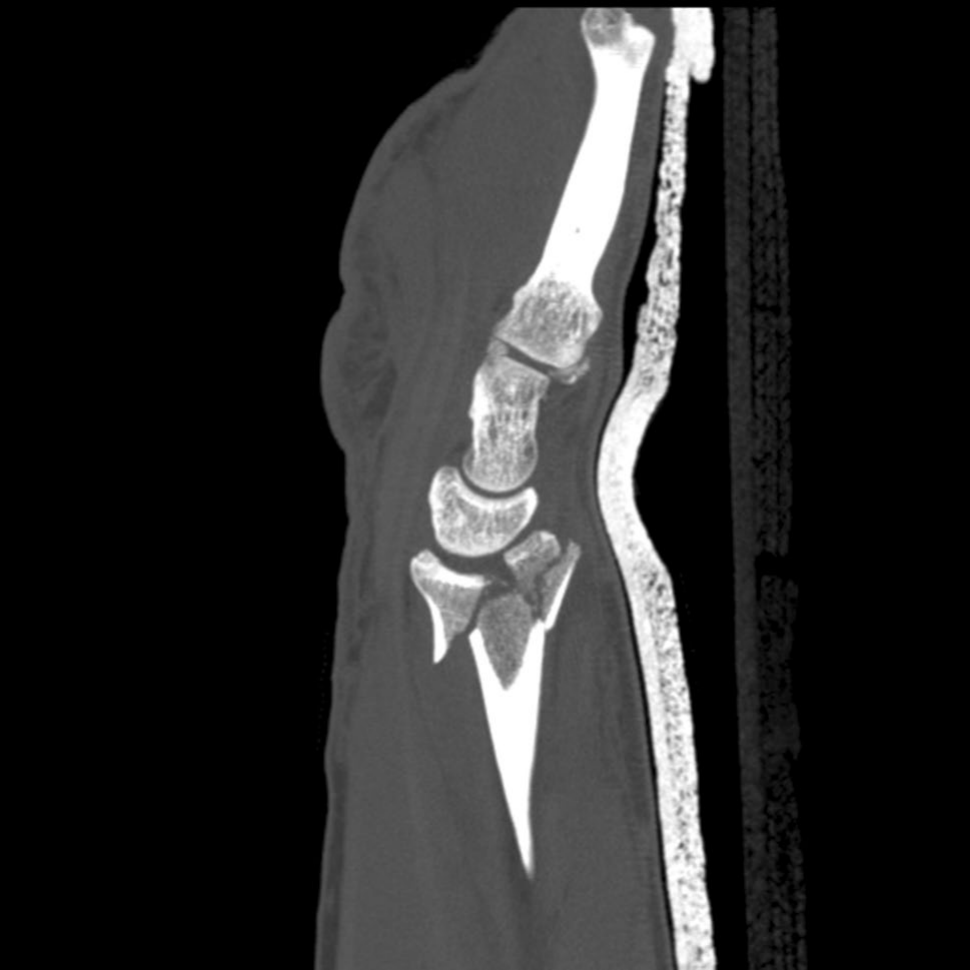
CT scan of a lateral view of the intermediate column showing the volar fragment of the intermediate column, a fracture gap and step of more than 2 mm and the volar fragment additionally toppled

**Fig. 14:**
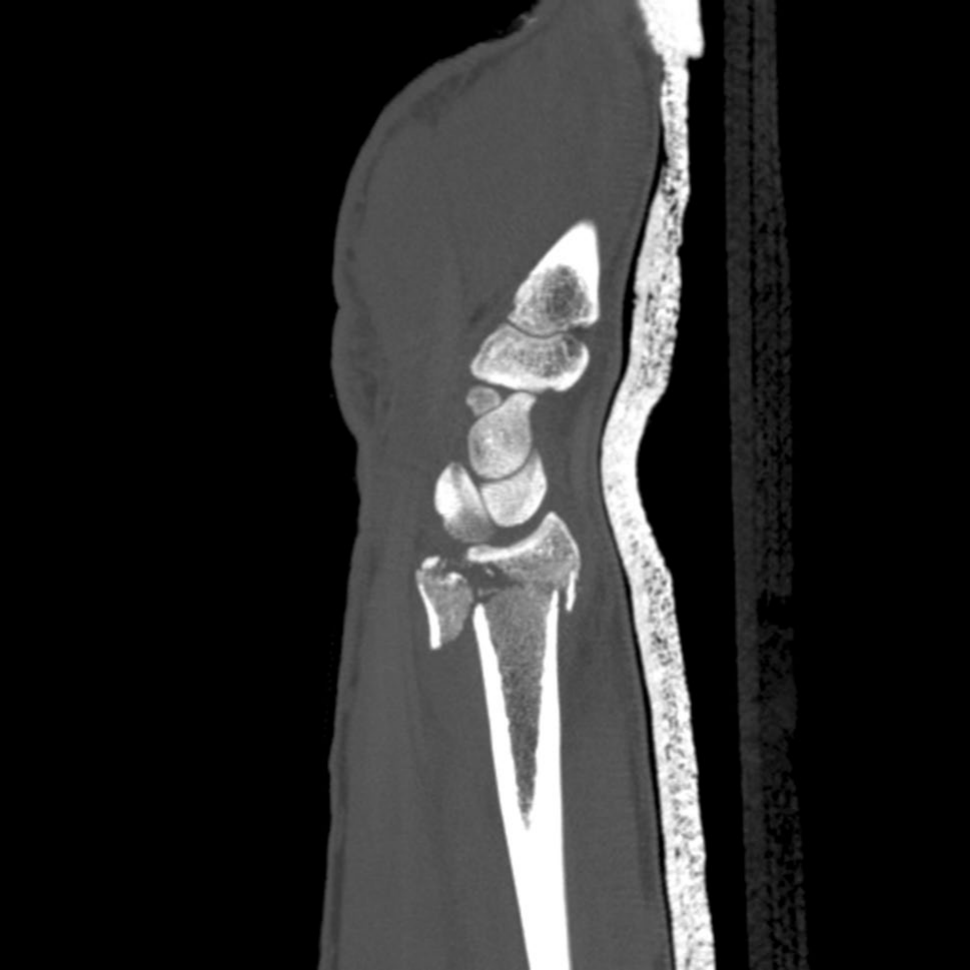
CT scan of a lateral view of the ulnar column showing the additional ulnar fragment flipped 90°

**Fig. 15:**
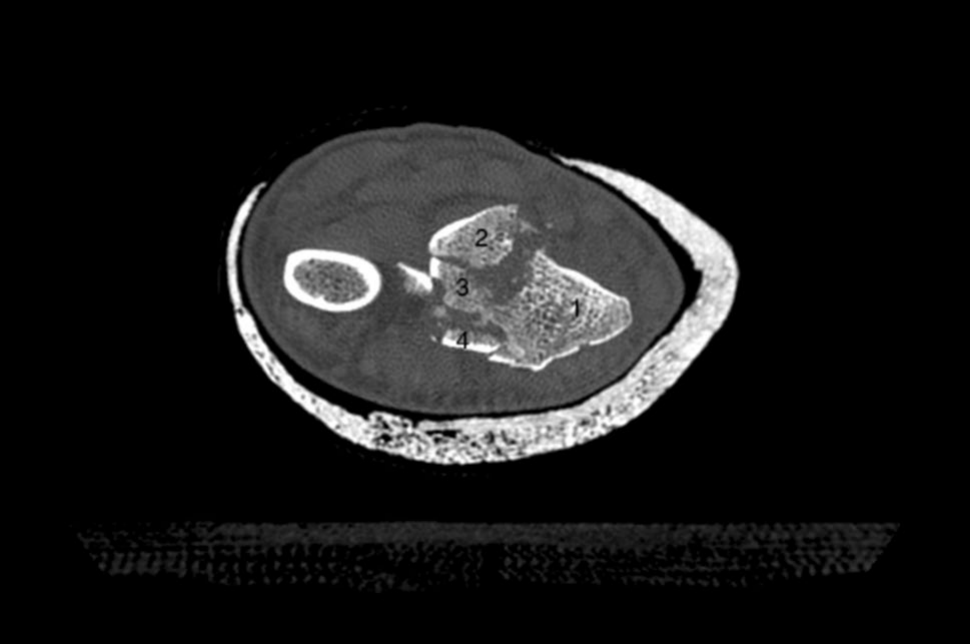
CT scan of an axial view with the numbered intraarticular fragments 1: radial styloid, 2: dorso-ulnar fragment, 3: fragment of the intermediate column 4: volar fragment

**Fig. 16:**
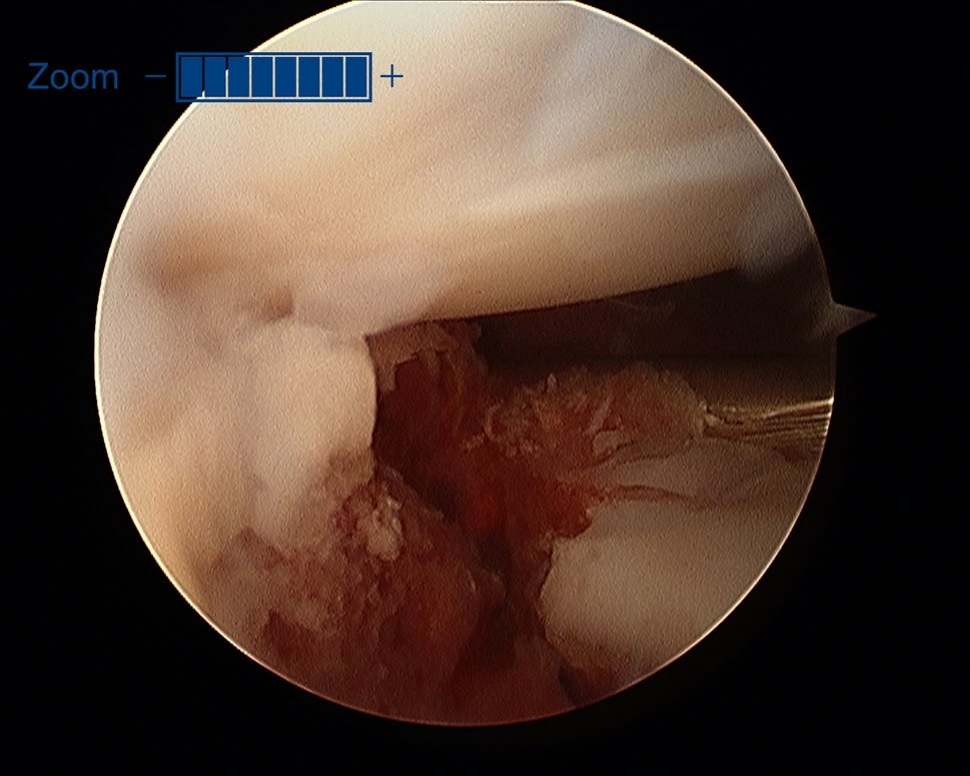
Initial view of the displaced fracture fragments after inserting the arthroscope through the 3/4 portal

**Fig. 17:**
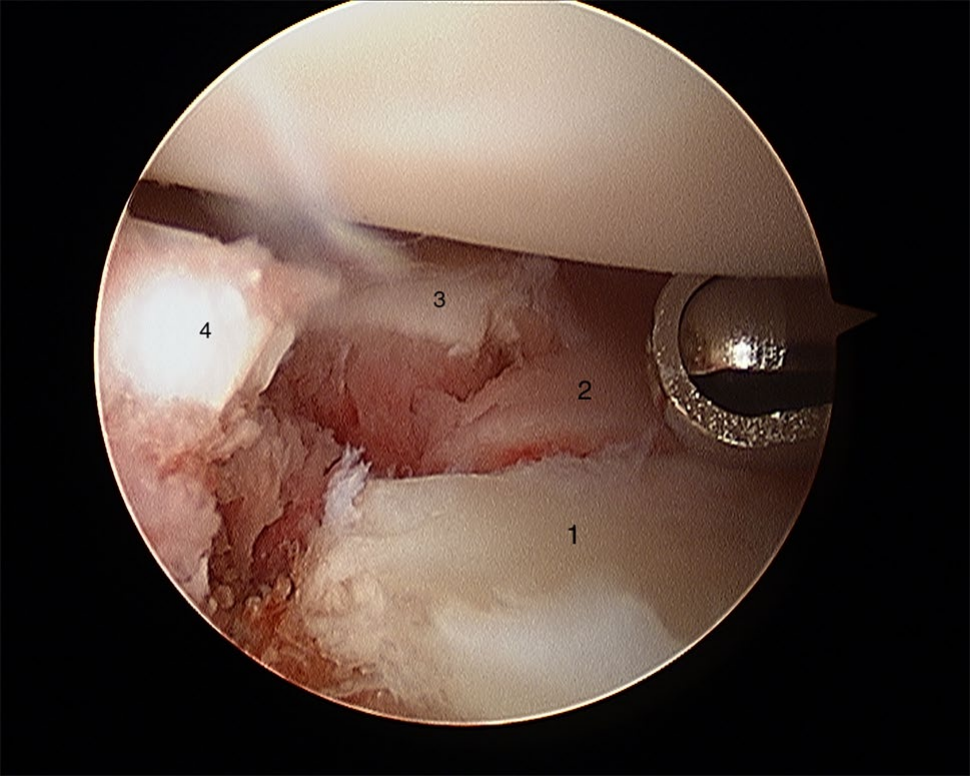
Arthroscopic view after debridement using the wrist shaver; all fragments are visualized. 1: radial fragment, 2: volar fragment, 3: intermediate fragment, 4: dorsal fragment

**Fig. 18:**
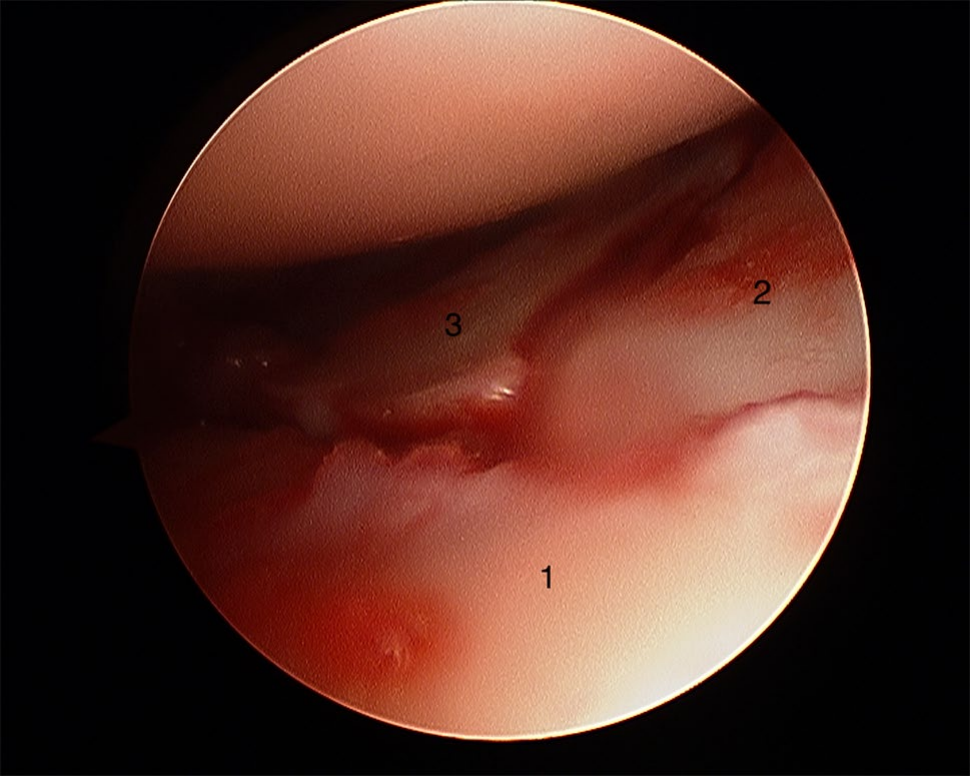
Arthroscopic view after arthroscopical fracture reduction showing an anatomical alignment of the involved fragments. 1: radial fragment, 2: volar fragment, 3: intermediate fragment

**Fig. 19:**
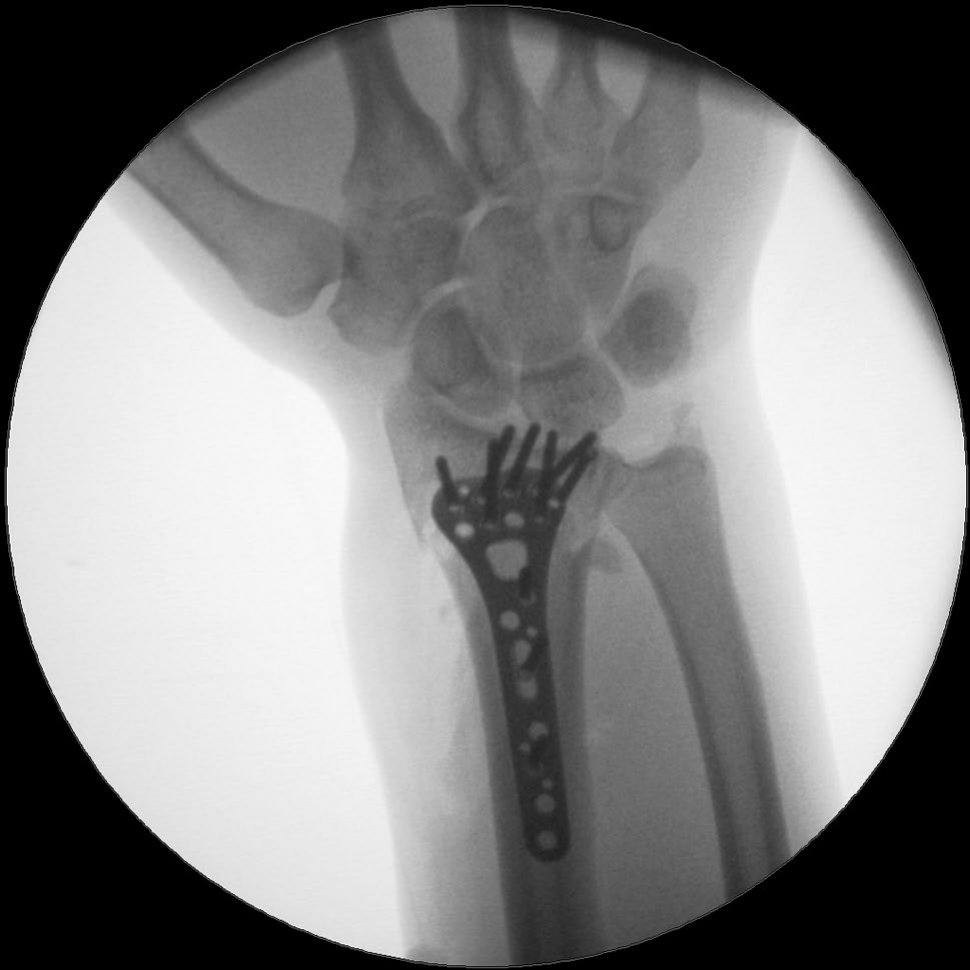
Dorsovolar fluoroscopic view at the end of the surgery showing the main screw fixation directed to the ulnar corner

**Fig. 20:**
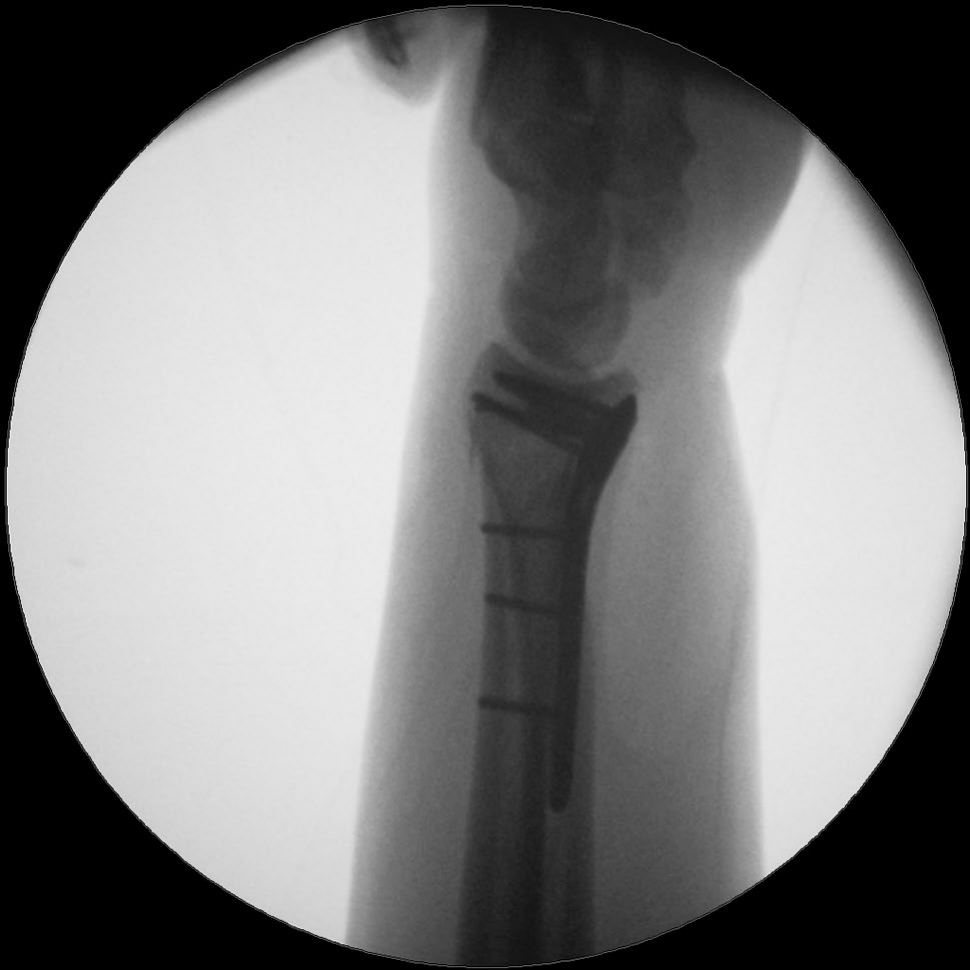
Lateral fluoroscopic view showing the screws in a subchondral position

**Fig. 21:**
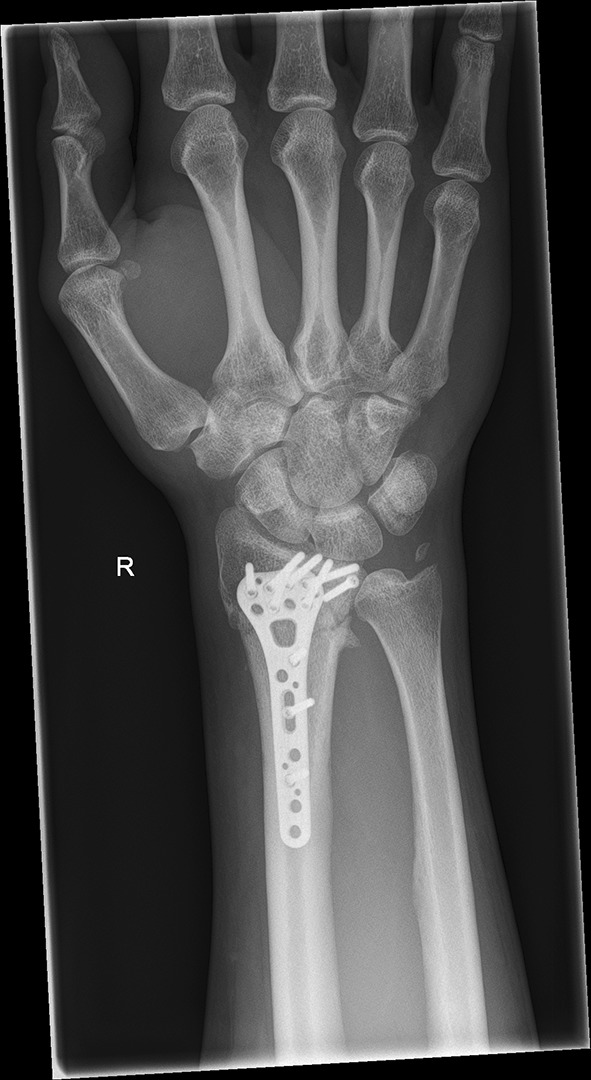
Dorsovolar and lateral x-ray control four weeks after surgery

**Fig. 22:**
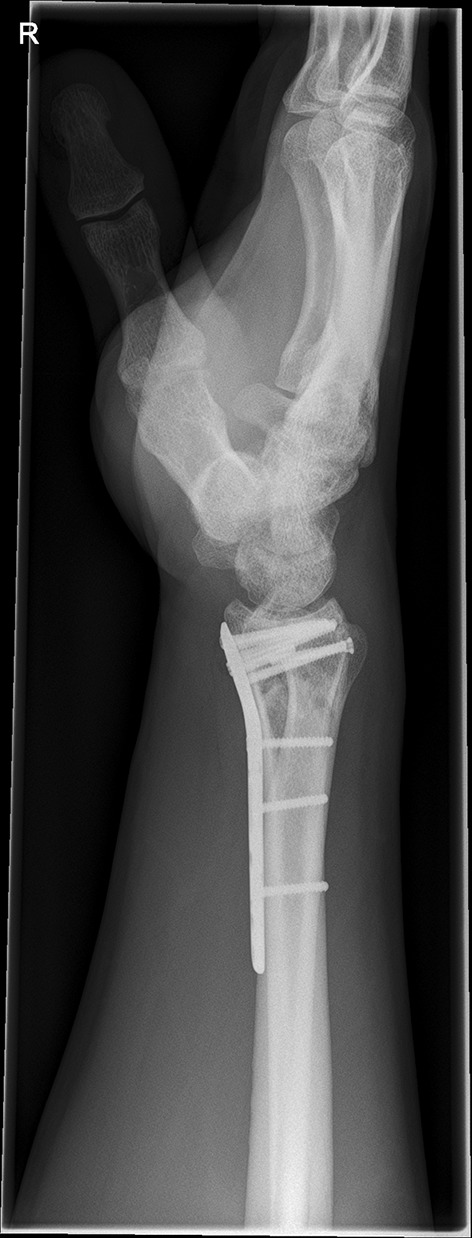
Dorsovolar and lateral X-ray control 4 weeks after surgery

**Fig. 23:**
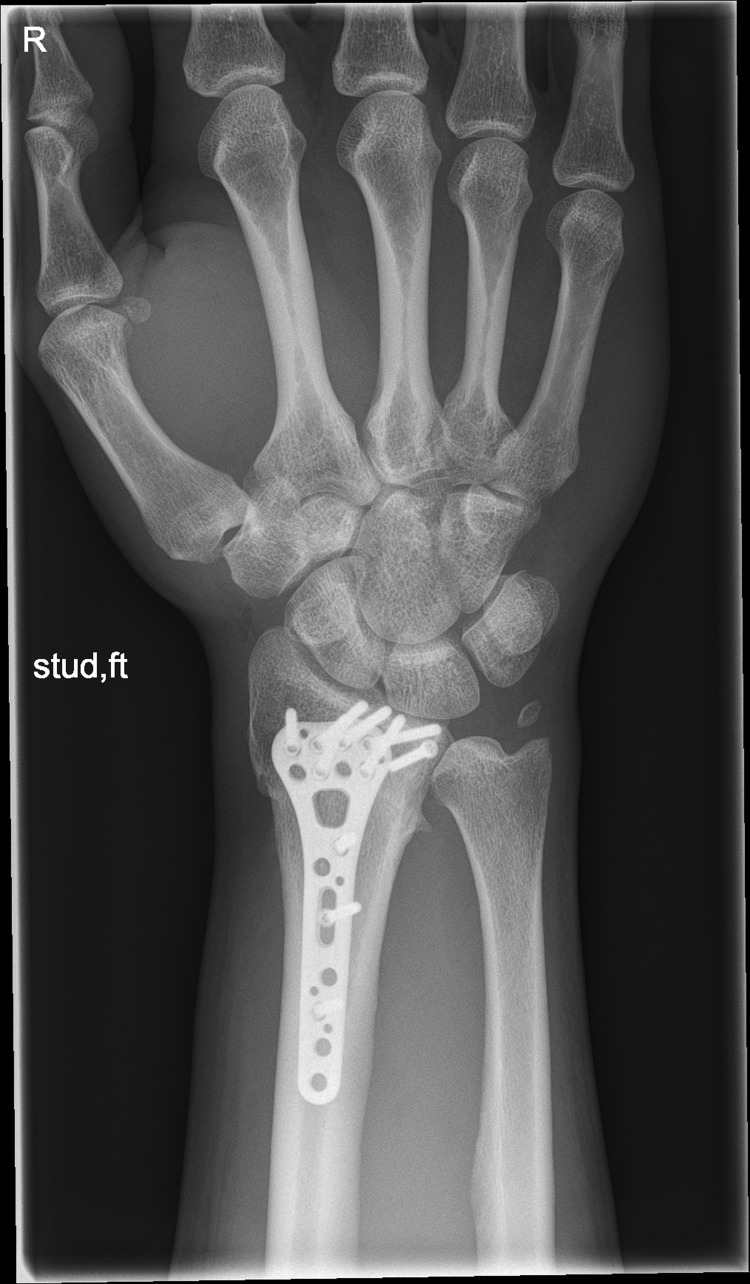
Dorsovolar and lateral X-ray control 1 year after surgery

**Fig. 24:**
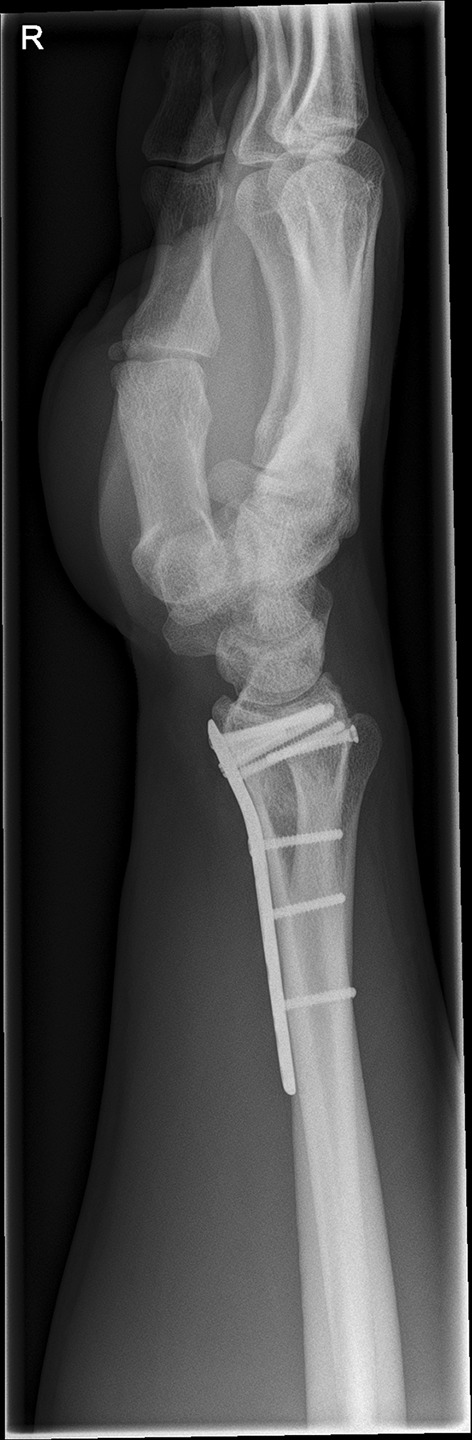
Dorsovolar and lateral X-ray control 1 year after surgery

**Fig. 25:**
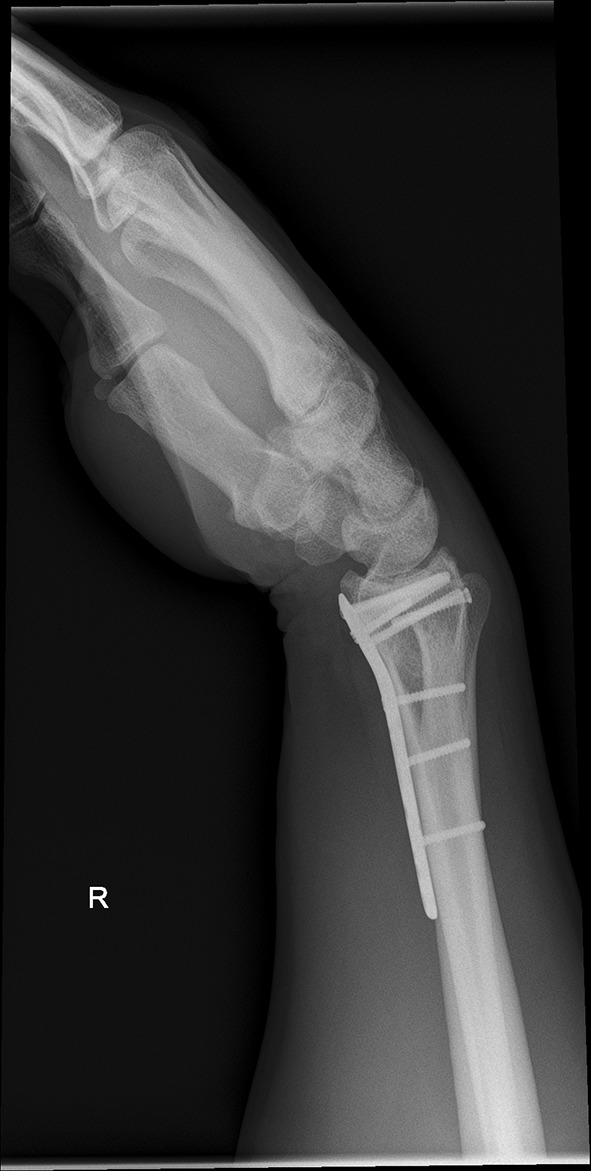
Lateral view in a flexed and extended position of the wrist before implant removal

**Fig. 26:**
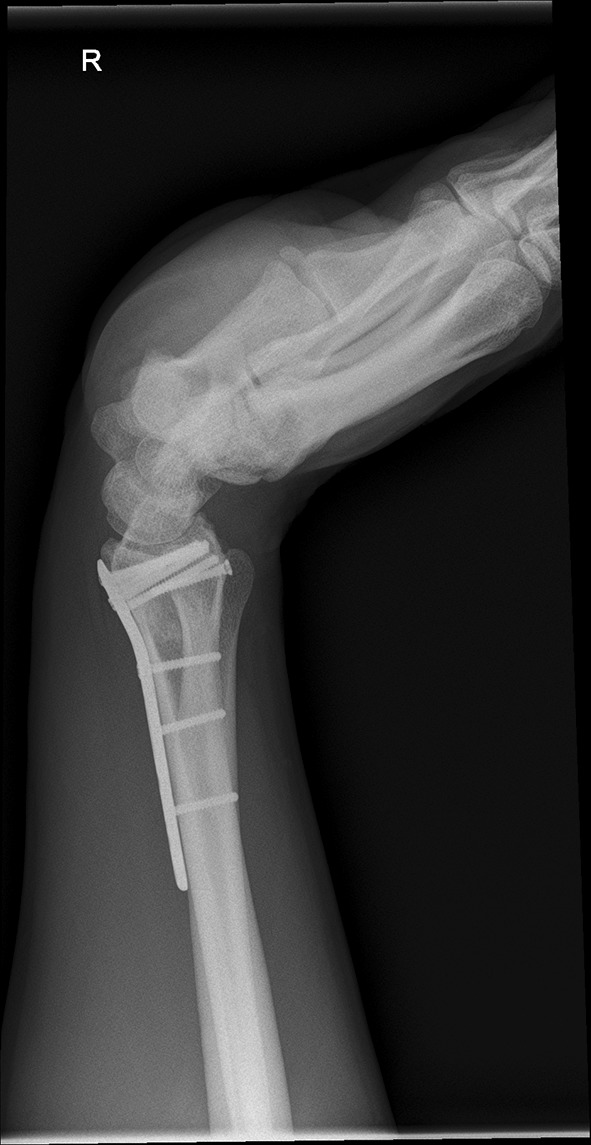
Lateral view in a flexed and extended position of the wrist before implant removal

**Fig. 27:**
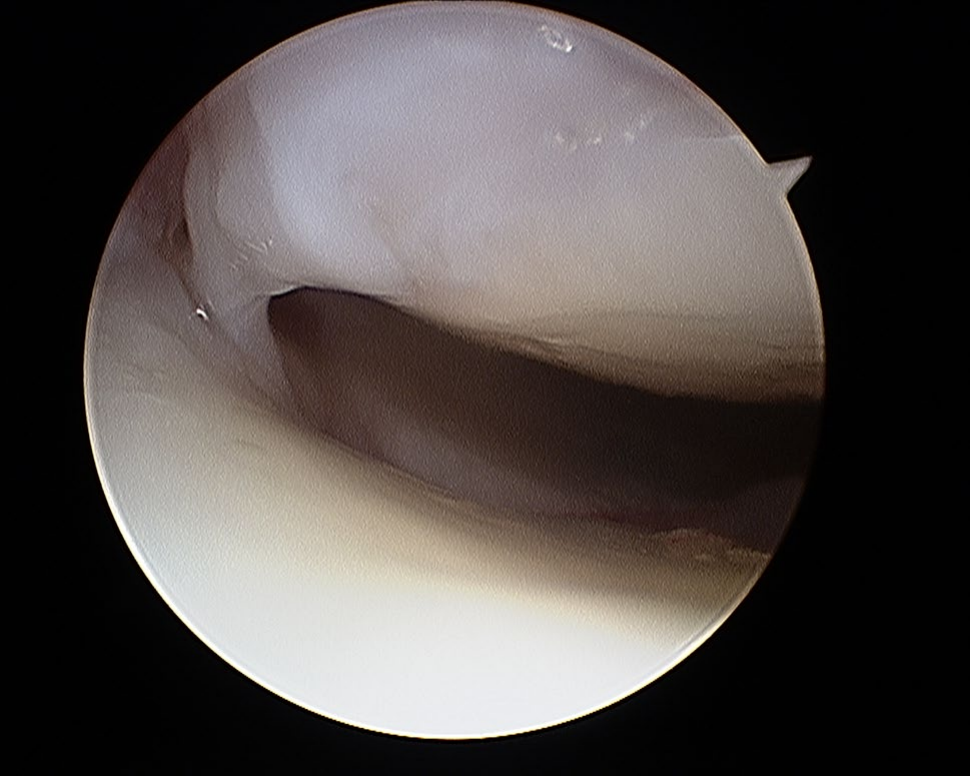
Arthroscopic view of the palmar side identifying palmar scar formations

**Fig. 28:**
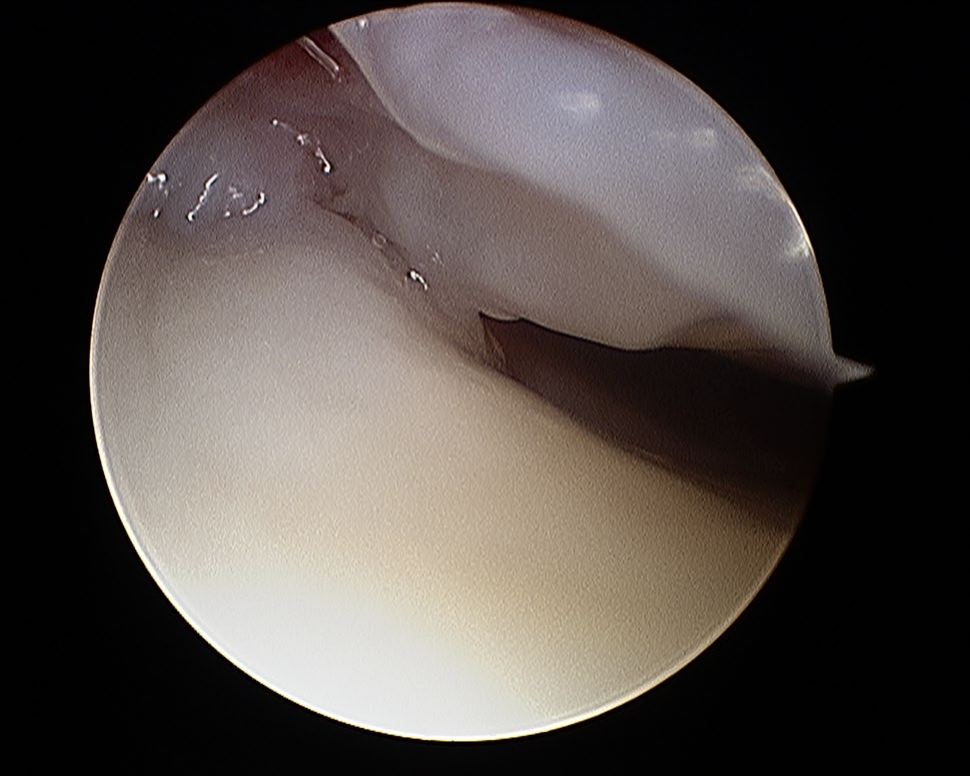
Arthroscopic view of the palmar side identifying palmar scar formations

**Fig. 29:**
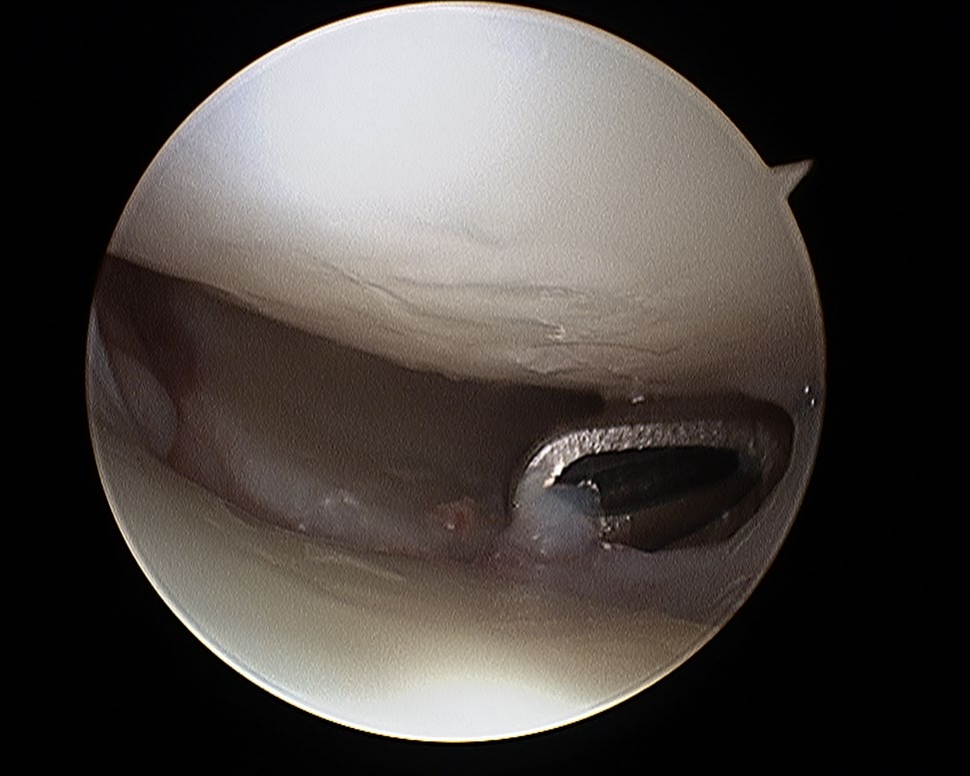
Arthroscopic view of the dorsal side identifying palmar scar formations

**Fig. 30:**
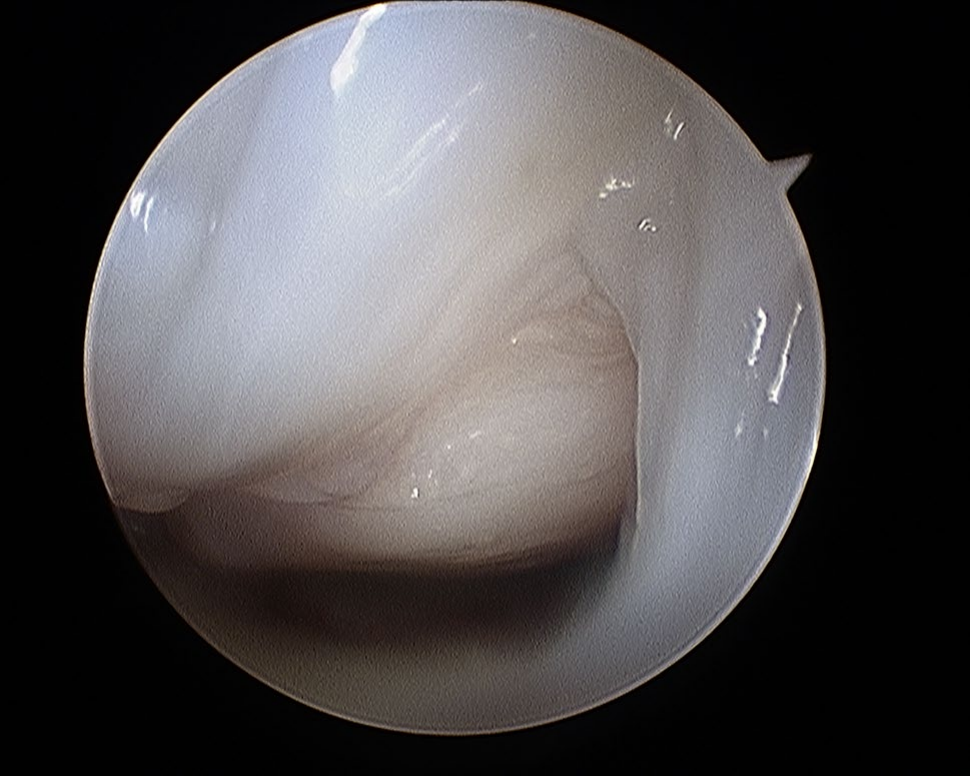
Arthroscopic view of the dorsal side identifying palmar scar formations

**Fig. 31:**
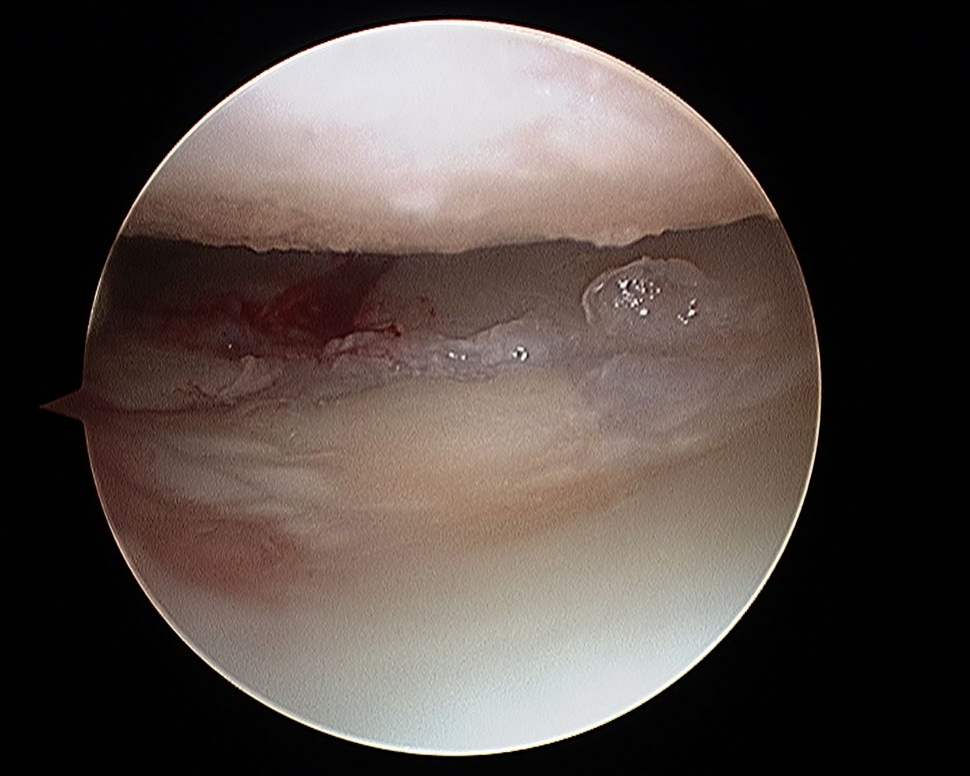
Arthroscopic view after debridement showing a gap at the volar side

**Fig. 32:**
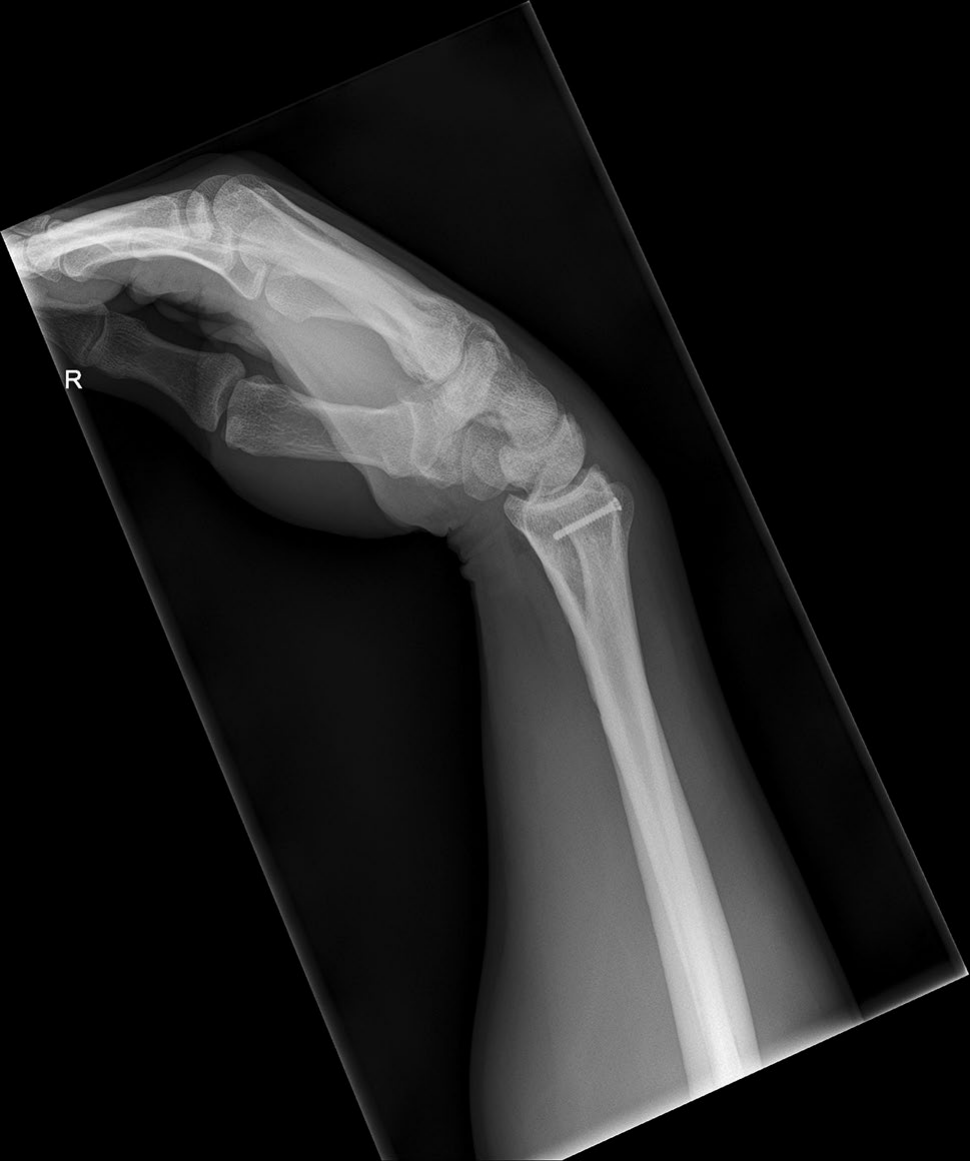
Lateral view in a flexed and extended position of the wrist after implant removal and arthroscopic debridement showing an improved range of motion

**Fig. 33:**
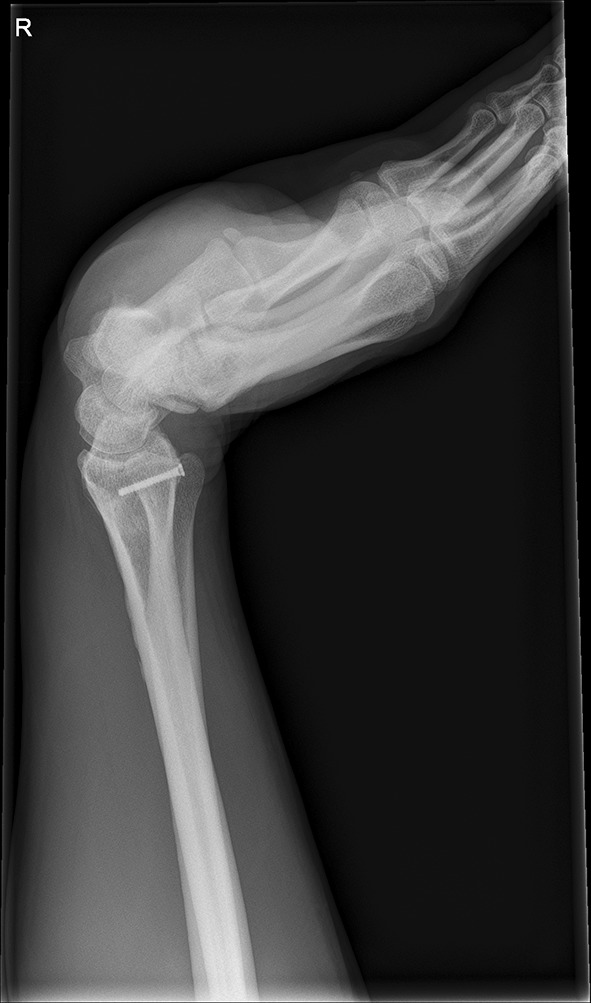
Lateral view in a flexed and extended position of the wrist after implant removal and arthroscopic debridement showing an improved range of motion

**Fig. 34:**
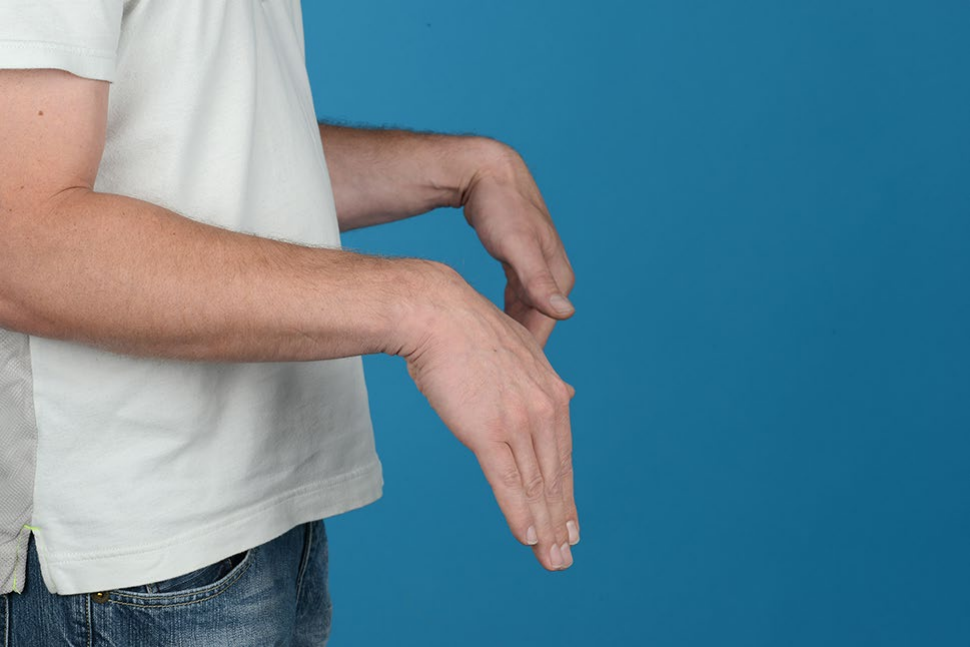
Clinical photographs in wrist extension and flexion after implant removal

**Fig. 35:**
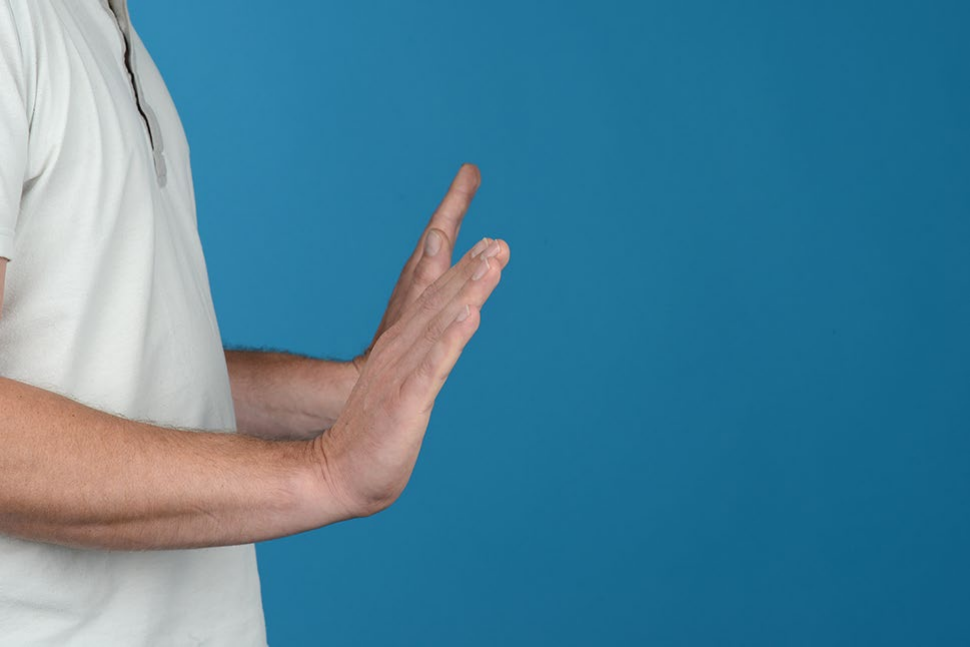
Clinical photographs in wrist extension and flexion after implant removal

Additionally, two palmarly located fragments and a dorso-ulnar fragment were detected in the CT scan performed after initial closed reduction and cast immobilization (Figs. 12, 13, 14 and 15). At the time of surgery, after plate presetting and insertion of the arthroscope through the portal 3/4, the displaced fracture fragments were evaluated (Fig. 16) and debrided (Fig. 17) using a shaver. After arthroscopically assisted fracture reduction, the fragments showed anatomical alignment (Fig. 18). The fluoroscopic images reassured the anatomic reduction in the antero-posterior and lateral view (Figs. 19 and 20). X-ray follow-up at 4 weeks after surgery showed a stable situation without any loss of reduction (Figs. 21 and 22). After 1 year X-rays showed a stable situation (Figs. 23 and 24). Occasional therapy was continuously performed until the range of motion (ROM) was S (Extension/Flexion) 60/0/40 and R (Rotation) 80/0/80 (Figs. 25 and 26). The arthroscopic view at the time of implant removal showed a palmar (Figs. 27 and 28) and dorsal (Figs. 29 and 30) scar formations limiting the ROM. Intra-articular debridement was conducted (Fig. 31). Eight months after implant removal X-rays in extended and flexed wrist position were conducted showing a ROM S (Extension/Flexion) 80/0/80 (Figs. 32 and 33). Clinical results of the patient in an extended und flexed position showing the good ROM (Figs. 34 and 35).
